# Microbial metabolites associated with healthy lifestyles in relation to metabolic syndrome and vascular health: a cross-sectional study

**DOI:** 10.1128/msystems.01433-25

**Published:** 2025-12-23

**Authors:** Zhuoyu Zhang, Bingqi Ye, Jialin He, Li Xiang, Siqi Li, Jiaqi Zhao, Wanlan Chen, Qi Zhang, Wanying Zhao, Jialu Yang, Yi Li, Jingmeng Ju, Yan Liu, Min Xia

**Affiliations:** 1 Guangdong Provincial Key Laboratory of Food, Nutrition and Health, and Department of Nutrition, School of Public Health, Sun Yat-sen University 26469 , Guangzhou, China; 2 Guangdong Provincial Key Laboratory of Food, Nutrition and Health, and Department of Statistics and Epidemiology, School of Public Health, Sun Yat-sen University 26469 , Guangzhou, China; University of Southampton, Southampton, United Kingdom

**Keywords:** gut microbiota, metabolic syndrome, healthy lifestyle, Mendelian randomization, betaine, cinnamoylglycine, vascular health

## Abstract

Lifestyle behaviors influence the risk of metabolic syndrome (MetS) and affect vascular health. However, the interactions between gut microbiota and lifestyle behaviors in relation to MetS, as well as the specific microbial taxa and metabolites involved, remain unclear. Here, we aimed to investigate the associations among healthy lifestyle behaviors, gut microbiota, and MetS and to explore the potential mediating roles of microbially derived metabolites in these associations. A total of 1,342 participants with complete assessments of the Healthy Lifestyle Score (HLS), MetS, and vascular health were enrolled. Fecal samples were collected and subjected to metagenomic sequencing. Host genetic data were obtained using a high-density genotyping array, and plasma metabolites were quantified by liquid chromatography–mass spectrometry. Using generalized linear models, we found that increased abundances of *Alistipes putredinis*, *Odoribacter splanchnicus*, and *Roseburia hominis* were associated with higher HLS and a reduced risk of MetS. Eleven microbial metabolic pathways were independently correlated with both HLS and MetS. Furthermore, increased plasma levels of cinnamoylglycine and betaine, driven by enhanced microbial capacity for homolactic fermentation, were identified as potential microbial effectors associated with MetS and vascular health. These findings indicate that the association between HLS and MetS may involve modulation of the gut microbiota and their metabolites and highlight the potential to enhance the beneficial effects of healthy behaviors on MetS and vascular health through microbiota-modifying interventions.

**IMPORTANCE:**

Metabolic syndrome raises the risk of heart disease and diabetes, yet practical levers to prevent it remain limited. We show that everyday healthy habits align with a gut microbial “signature” linked to better vascular health and lower metabolic risk. Using metagenomics, metabolomics, and genetic causal analyses, we identify specific bacteria (*Alistipes putredinis*, *Odoribacter splanchnicus*, and *Roseburia hominis*) and microbially produced molecules—especially cinnamoylglycine and betaine from enhanced homolactic fermentation—that may mediate these benefits. These findings connect lifestyle, the gut microbiome, and blood metabolites in a single framework, suggesting actionable biomarkers to monitor risk and potential microbiota-targeted strategies (diet and pre/probiotics) to improve cardiometabolic health. By highlighting concrete microbial pathways and metabolites, our work advances the path toward precision prevention and low-cost interventions for metabolic syndrome and vascular disease.

## INTRODUCTION

Metabolic syndrome (MetS) is characterized by a combination of several metabolic disorders including central obesity, hypertension, dyslipidemia, and hyperglycemia. Generally, the prevalence of MetS is estimated as 34.7% in the United States (1) and 33.9% in China (2), and it has risen in recent decades (3). Moreover, MetS is characterized by low-grade inflammation that adversely affects vascular health (4). Studies have demonstrated a significant association between metabolic syndrome and early vascular pathologies, including peripheral arterial disease (PAD), arterial stiffness, and increased intima-media thickness (IMT) (5–7). Lifestyle modifications are widely regarded as the most effective preventive strategies against MetS and cardiovascular disease (CVD), with exercise interventions and dietary adjustments often producing substantial benefits (8). However, older adults frequently face physical limitations that prevent them from meeting recommended activity levels, and long-term dietary changes can be constrained by socioeconomic and environmental factors. Consequently, there is a pressing need to explore more accessible and direct preventive approaches.

The gut microbiome has recently emerged as an important regulator of host metabolism and the development of various chronic diseases. Indeed, accumulating studies suggest a causal relationship between gut microbiota dysfunction and the pathogenesis of MetS and CVD (8–10). Alterations in the gut microbiota, such as a higher *Firmicutes-to-Bacteroidetes* ratio and a decrease in the relative abundance of *Peptostreptococcaceae* and Turicibacter families, are associated with MetS (11). In a human study, transferring a healthy lean donor’s microbiome into an obese recipient with MetS markedly increased insulin sensitivity within 6 weeks (12, 13). Furthermore, certain species, including *Prevotella, Lactobacillus*, and *Bacteroides*, are associated with insulin resistance, a hallmark of MetS (13–15). On the other hand, species such as *Akkermansia muciniphila* and *Faecalibacterium prausnitzii* are linked to increased insulin sensitivity (16, 17). Additionally, *Akkermansia* has been found to have a strong association with CVD and improve atherosclerosis in mice (18, 19). Notably, the impacts of gut microbiota and their metabolites are not limited to influencing MetS; they also play crucial roles in cardiovascular health. Various microbial species and their metabolic byproducts can affect endothelial function, contributing to arterial stiffness and atherosclerosis (20). Importantly, lifestyle factors also shape the gut microbiome, offering avenues to modify microbial ecology and improve cardiometabolic health. A human study revealed that consuming fried meat reduced microbiota diversity, decreased the abundances of *Lachnospiraceae* and *Flavonifractor*, and increased the abundances of *Dialister*, *Dorea*, and *Veillonella* (21). Exercise has also been found to alter gut microbiota composition and function in both lean and obese individuals (22). In recent years, numerous studies have shown that microbial metabolites are the functional effectors through which alterations in the gut microbiota influence disease (23, 24). Gut microbiota-derived hippuric acid has been reported to alleviate hyperuricemia by upregulating the expression of the urate transporter ABCG2 and promoting its localization to the intestinal brush border membrane, thereby enhancing intestinal urate excretion (25). Additionally, *Flavonifractor plautii*-derived *cis-aconitic acid* has been shown to protect against arterial stiffness by improving the elastic fiber network and reducing pulse wave velocity, a protective effect mediated through the suppression of matrix metalloproteinase-2 (MMP-2) and the inhibition of monocyte chemoattractant protein-1 (MCP-1) and nuclear factor kappa-B (NF-κB) activation (20). Similarly, we observed that exercise is associated with shifts in gut microbial functional pathways, including amino acid biosynthesis and carbohydrate metabolism (26). In addition, cigarette smoking has been linked to gut dysbiosis and downstream alterations in bile acid metabolites (27). Together, these findings support the concept that lifestyle behaviors can modulate the gut microbiota and its metabolic functions. However, whether and how alterations in gut microbiota are functionally involved in the adverse effect of healthy lifestyle factors on MetS and vascular health remain obscure.

Here, to elucidate how gut microbial alterations mediate the effect of HLS on MetS and vascular health, we conducted an integrative multiomics analysis, including gut microbiota, host genetics, and plasma metabolites, in a cohort of participants from China.

## MATERIALS AND METHODS

### Study participants

This study was approved by the Ethics Committee of School of Public Health, Sun Yat-Sen University (L2017-001) and was in accordance with the principles of the Declaration of Helsinki. Written informed consents were obtained from each individual. Local residents, who have lived in Guangdong Province, China, for over 5 years, were invited for health screening through flyers and advertisement at the Community Healthcare Center of Chashan Town (Dongguan City, Guangdong, China) (28). Fecal metagenomics and plasma metabolomics data were available from 1,423 participants aged between 35 and 78 years. Inclusion criteria were as follows: (i) absence of severe disability, any malignant tumors, thyroid disorder, biliary acute or chronic viral hepatitis, cirrhosis, chronic renal insufficiency, acute or chronic inflammatory disease; (ii) not pregnant; and (iii) able to understand the nature and possible consequence of the study. Exclusion criteria were as follows: (i) use of antibiotics within 3 months at sample collection and (ii) inadequate information for the assessment of metabolic syndrome. Finally, a total of 1,342 participants were included in this analysis (Fig. S1). More details about the collection of metadata and biological samples are provided in the supplemental material.

### Definition of HLS

Lifestyle factors were assessed by trained staff via structured questionnaires, which are detailed in the supplemental material. An HLS based on cigarette smoking, drinking, physical activity, diet, and body shape was constructed as described (29). Detailed HLS definitions are provided in the supplemental methods. All five components were summed to obtain the HLS, ranging from 0 to 5, with a higher score indicating a healthier lifestyle.

### Assessment of MetS

According to the National Cholesterol Education Program-Adult Treatment Panel III (NCEP-ATP III) (30), MetS was defined as having three or more metabolic disorders: (i) central obesity, defined as waist circumference ≥90 cm in males, or ≥80 cm in females; (ii) elevated blood pressure, defined as SBP ≥130 mmHg, or DBP≥85 mmHg, or treatment of previously diagnosed hypertension; (iii) elevated triglycerides (TG), defined as having a fasting TG ≥1.7 mmol/L; (iv) reduced high-density lipoprotein cholesterol (HDL-c), defined as having HDL-c <1.03 mmol/L in males, or <1.30 mmol/L in females; and (v) elevated fasting glucose, defined as having a fasting glucose ≥5.6 mmol/L, or use of any hypoglycemic drugs.

### Measurement of brachial-ankle pulse wave velocity (baPWV) in study participants

The baPWV was simultaneously measured using an automatic waveform analyzer (BP-203 PRE III, Omron Health Medical, Dalian, China), as previously described (20). Subjects with elevated arterial stiffness were defined as those with a baPWV value ≥ 1,400 cm/s (20).

Measurements were conducted in a room with a controlled temperature of 22°C–25°C. After a minimum rest period of 5 min in the supine position, four cuffs were wrapped around the upper arms and ankles and connected to a plethysmographic sensor (for volume pulse) and an oscillometric pressure sensor. The baPWV was calculated using the following formula: baPWV = (La - Lb) / Tba, where La is the distance from the heart to the ankle, Lb is the distance from the heart to the brachium, and Tba is the transmission time between the brachial and posterior tibial artery waveforms.

To minimize bias in baPWV measurements in patients with severe atherosclerosis in the lower extremities, subjects with a bilateral ankle-brachial index (ABI) < 0.9 or substantial side differences in baPWV (more than 1,000 cm/s) were excluded from the analysis. For subjects with unilateral ABI < 0.9, only the baPWV of the unaffected side was considered as the final reading. Otherwise, the average value of the left and right baPWV was used as the final reading, as previously described. Right and left ABIs were calculated as the ratio of the higher of two systolic pressures in the lower limbs (posterior and anterior tibial arteries) to the control brachial systolic pressures. The lowest of the values obtained was used for analysis. PAD was operationally defined as an ABI ≤0.90 or ≥1.40 (31).

### Carotid ultrasound examination

Carotid ultrasound examinations were performed by professionally trained sonographers using a high-resolution ultrasound device, the Toshiba Aplio 400. Participants were positioned in the supine position with their heads slightly tilted backward and rotated to the contralateral side to fully expose the carotid arteries. The examination included longitudinal and transverse scans of the bilateral common carotid arteries (CCA), carotid bifurcation, internal carotid arteries, and external carotid arteries. Carotid IMT was measured on the posterior wall of the distal 1 cm segment of the CCA, and the average of three measurements was recorded. Early arterial lesions were defined as a focal thickening of the IMT (≥0.9 mm) or plaque. Left and right common carotid IMT were obtained for each participant, and the mean was considered in the analysis. All ultrasound images were independently analyzed by two observers, and in cases of disagreement, a senior physician provided the final assessment (32).

### Fecal DNA extraction and shotgun metagenomic sequencing

Participants received the MGIEasy stool collection kit containing a room temperature stabilizing reagent and detailed instructions at the community center during study visit. Stool samples were collected on the same day of blood drawing and stored at −20°C at the community center for a maximum of 1 day before transportation to the central freezer at −80°C until analysis. Stool DNA was extracted from frozen stools using the MagMAXTM Microbiome Ultra Nucleic Acid Isolation kit (Thermo Fisher Scientific, MA, USA). Gut microbiota were profiled by whole-genome shotgun metagenomic sequencing. All samples were sequenced on the Illumina NovaSeq 6000 platform (Illumina, San Diego, California, USA; paired-end; insert size, 350 bp; read length, 150 bp) by Novogene Co., Ltd (Beijing, China). Taxonomic profiling was performed using MetaPhlAn3 (v3.0.13) with default parameters. All taxa data were reported as relative abundances (total sum scaling normalization) in each sample for subsequent analysis. More details about quality control and data analysis for metagenomics are provided in the supplemental methods.

### Human genomics and bidirectional Mendelian randomization (MR) analysis

Host DNA was extracted from buffy coat using TIANamp Blood DNA Kit from TIANGEN Co., Ltd (Beijing, China) according to the manufacturer’s instruction. The dosage of DNA used for subsequent library preparation was more than 1 μg, and their concentration was controlled at no less than 80 ng/μL. Genotyping was performed with Infinium Chinese Genotyping Array-24 v1.0 BeadChip at Illumina platform by WeGene Co., Ltd (Shenzhen, China). More details about quality control and data analysis are provided in the supplemental methods. To explore host genetic variants linked to gut microbiota or microbial metabolites, a threshold of *P* < 5 × 10^−5^ was adopted to maximize explained genetic variance, as previously described (33). To investigate the associations between microbial species and MetS, bidirectional MR analysis was performed using the MendelianRandomization R package (version 0.10.0), with taxa relative abundances as exposures and binary MetS as the outcome to evaluate potential causal links. MR estimates were evaluated by three methods, including inverse-variance weighted (IVW), simple median, and MR-Egger regression. The IVW method served as the primary estimator, aggregating SNP-specific Wald ratios with IVWs (fixed-effect meta-analysis) to provide an efficient estimate, assuming valid instruments and no directional pleiotropy. More details are provided in the supplemental methods.

### Metabolomics profiling and targeted quantification of key metabolites

#### Untargeted metabolomic profiling

After at least 10 h of fasting, blood samples were collected and stored at −80°C for further analysis. An integrated approach for large-scale detection, identification, and quantification of widely targeted metabolites was performed by Metware (Wuhan, China), as previously described (34, 35).

#### Targeted metabolite quantification

After performing differential and correlation analyses, combined with biological validation, we identified betaine and cinnamoylglycine as key metabolites. To further confirm their concentration levels, we subsequently performed a targeted quantitative analysis using ultra-performance liquid chromatography–tandem mass spectrometry (UPLC–MS/MS) with isotope-labeled internal standards (betaine-d9 and [2,2-²H₂]-N-trans-cinnamoylglycine, CMG-d₂), enabling absolute quantification of these metabolites in plasma.

More details concerning metabolomics profiling and data analysis, as well as targeted metabolomics analysis, are provided in the supplemental methods. Metabolomics results are available in Tables S6 through S8.

### Statistical analysis

All statistical analyses were performed using R software version 4.3.0 (R Foundation for Statistical Computing, Vienna, Austria). Continuous parameters were examined for normality using the Shapiro-Wilk test. Since all continuous variables were nonnormally distributed, they were presented as median with interquartile range (IQRs) and analyzed with Wilcoxon rank-sum test. Categorical variables were presented as counts and percentages (%) and analyzed with χ^2^ test or Fisher exact test, as appropriate. To assess nonlinear associations between plasma metabolites and CVD, we fitted restricted cubic-spline (RCS) models with the rcssci package. Predictive performance was then gaged with receiver-operating-characteristic (ROC) curves. Full methodological details are provided in the supplemental methods.

## RESULTS

### Basic characteristics of study participants

**Fig 1:**
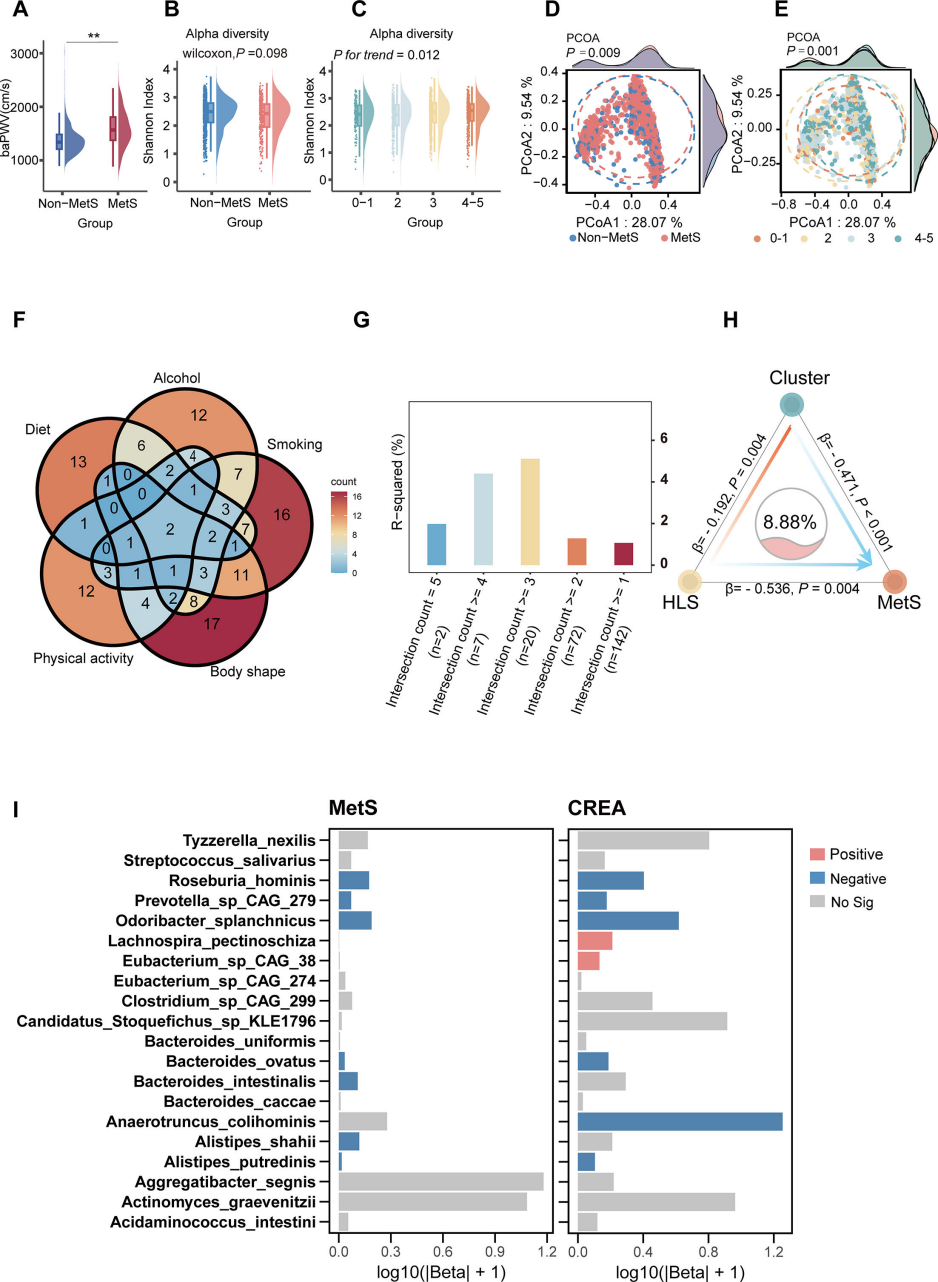
Microbial species associated with HLS and MetS. (**A**) baPWV levels in subjects with and without MetS (*P* values were determined by Wilcoxon rank-sum test, with ^*^P < 0.05, ^**^P < 0.01, and ^***^P < 0.001). (**B and C**) Alpha diversity of gut microbiota in subjects stratified by MetS (**B**) and across different HLS (**C**). (**D and E**) Beta diversity of gut microbiota in subjects stratified by MetS (**D**) and across different HLS groups (**E**). (**F**) Venn diagram showing the overlap of species significantly associated with five lifestyle factors: body shape, diet, physical activity, smoking, and alcohol. (**G**) Bar plot illustrating the model fitting (R^2^) values, representing the variance in MetS explained by the PCoA1 values of gut microbiota. (**H**) Cluster mediation analysis depicting the relationships between HLS, gut microbiota clusters, and MetS, adjusted for age, sex, education level, medication use, and family medical history. (**I**) Independent associations of 20 selected bacterial species with MetS and creatinine (CREA), adjusted for age, sex, education level, medication use, and family medical history. Red, blue, and gray bars indicate positive (*P*_adj_ values were determined by linear regression, with *P*_adj_ < 0.05), negative (*P*_adj_ values were determined by logistic regression, with *P*_adj_ < 0.05), and insignificant correlations with MetS, respectively.

The baseline characteristics of the 1,342 enrolled participants are summarized in Table 1. The cohort had a median age of 50 years (IQR: 45–56 years), and a median BMI of 23.91 kg/m² (IQR: 21.71–26.14 kg/m²), and consisted of 45.2% males. Demographically, there was a stepwise decrease across the groups in the proportions of MetS, hypertension, arterial stiffness, and early arterial lesions (*P* < 0.001). Participants adhering to 4 or 5 HLS were more likely to be female and to have a higher education level, lower BMI, lower alcohol intake, and greater dietary quality. Furthermore, baPWV was significantly higher in the MetS group than in the non-MetS group (*P* <0.001, Fig. 1A).

**TABLE 1:** Basic characteristics of the participants according to HLS and MetS^*a*^^,^^*b*^^,^^*c*^

Parameter	HLS = 0/1 (*N* = 149)	HLS = 2 (*N* = 406)	HLS = 3 (*N* = 494)	HLS = 4/5 (*N* = 293)	Total (*N* = 1,342)	*P* value	Non-MetS (*N* = 1,008)	MetS (*N* = 334)	*P* value
Female, *n* (%)	3 (2.01)	204 (50.25)	309 (62.55)	219 (74.74)	735 (54.77)	<0.001	578 (57.34)	157 (47.01)	0.001
Age, year	51.00 (46.00–57.00)	52.00 (46.00–57.00)	50.00 (44.00–57.00)	48.00 (43.00–55.00)	50.00 (45.00–56.00)	0.001	50.00 (44.00–56.00)	52.00 (46.00–59.00)	<0.001
Education									
Below high school, *n* (%)	107 (71.81)	306 (75.37)	341 (69.03)	191 (65.19)	945 (70.42)	0.026	683 (67.76)	262 (78.44)	<0.001
High school and above, *n* (%)	42 (28.19)	100 (24.63)	153 (30.97)	102 (34.81)	397 (29.58)		325 (32.24)	72 (21.56)	
BMI, kg/m^2^	25.53 (23.87–27.31)	25.07 (22.88–27.35)	23.63 (21.49–25.71)	22.31 (20.85–23.90)	23.91 (21.71–26.14)	<0.001	23.19 (21.16–25.14)	26.23 (24.56–28.24)	<0.001
Waist circumference, cm	90.50 (87.00–96.20)	87.75 (81.80–92.70)	82.05 (75.70–89.20)	76.70 (73.10–83.30)	84.00 (76.80–91.00)	<0.001	81.10 (75.05–88.15)	91.40 (86.00–95.60)	<0.001
Hip circumference, cm	97.10 (93.00–101.30)	96.10 (91.20–100.90)	94.20 (90.00–98.10)	92.90 (89.60–96.00)	94.50 (90.60–99.00)	<0.001	93.20 (89.60–97.40)	98.15 (94.50–102.50)	<0.001
WHR	0.93 (0.90–0.96)	0.91 (0.87–0.94)	0.88 (0.83–0.92)	0.84 (0.80–0.88)	0.89 (0.84–0.93)	<0.001	0.87 (0.82–0.91)	0.93 (0.89–0.96)	<0.001
Comorbidities									
Diabetes, *n* (%)	10 (6.71)	23 (5.67)	23 (4.66)	8 (2.73)	64 (4.77)	0.199	16 (1.59)	48 (14.37)	<0.001
Hypertension, *n* (%)	32 (21.48)	88 (21.67)	76 (15.38)	31 (10.58)	227 (16.92)	<0.001	114 (11.31)	113 (33.83)	<0.001
MetS, *n* (%)	56 (37.58)	143 (35.22)	100 (20.24)	35 (11.95)	334 (24.89)	<0.001	–	–	–
Medication									
Antidiabetic, *n* (%)	8 (5.37)	22 (5.42)	17 (3.44)	7 (2.39)	54 (4.02)	0.157	12 (1.19)	42 (12.57)	<0.001
Lipid lowering, *n* (%)	8 (5.37)	16 (3.94)	13 (2.63)	5 (1.71)	42 (3.13)	0.127	21 (2.08)	21 (6.29)	<0.001
Antihypertensive, *n* (%)	28 (18.79)	79 (19.46)	67 (13.56)	27 (9.22)	201 (14.98)	<0.001	97 (9.62)	104 (31.14)	<0.001
Healthy lifestyle factors									
Smoking status									
Never, *n* (%)	16 (10.74)	297 (73.15)	443 (89.68)	283 (96.59)	1039 (77.42)		796 (78.97)	243 (72.75)	0.001
Daily, *n* (%)	93 (62.42)	75 (18.47)	36 (7.29)	4 (1.37)	208 (15.50)	<0.001	156 (15.48)	52 (15.57)	
Former, *n* (%)	40 (26.85)	34 (8.37)	15 (3.04)	6 (2.05)	95 (7.08)		56 (5.56)	39 (11.68)	
Good diet quality, *n* (%)	8 (5.37)	53 (13.05)	221 (44.74)	236 (80.55)	518 (38.60)	<0.001	398 (39.48)	120 (35.93)	0.247
High physical activity, *n* (%)	11 (7.38)	47 (11.58)	165 (33.40)	212 (72.35)	435 (32.41)	<0.001	336 (33.33)	99 (29.64)	0.211
No heavy drinking, *n* (%)	96 (64.43)	378 (93.10)	472 (95.55)	289 (98.63)	1235 (92.03)	<0.001	929 (92.16)	306 (91.62)	0.75
Laboratory measures									
SBP, mm Hg	128.00 (119.50–140.00)	128.00 (117.50–140.00)	122.00 (113.00–134.00)	119.00 (109.50–130.50)	124.00 (114.00–136.00)	<0.001	120.50 (111.50–131.25)	134.50 (124.50–144.50)	<0.001
DBP, mm Hg	85.00 (79.00–92.50)	82.00 (75.50–90.50)	79.00 (73.50–86.00)	79.00 (72.00–85.50)	80.50 (74.00–88.00)	<0.001	79.00 (72.50–85.00)	86.00 (79.50–93.00)	<0.001
TG, mmol/L	1.58 (1.10–2.31)	1.33 (0.95–1.83)	1.10 (0.79–1.64)	0.98 (0.72–1.40)	1.18 (0.84–1.70)	<0.001	1.02 (0.76–1.40)	2.06 (1.45–2.84)	<0.001
TC, mmol/L	5.26 (4.82–5.83)	5.34 (4.71–6.10)	5.25 (4.73–5.89)	5.19 (4.61–5.93)	5.27 (4.67–5.94)	0.273	5.23 (4.66–5.90)	5.36 (4.84–6.10)	0.033
HDL-c, mmol/L	1.19 (1.00–1.35)	1.31 (1.10–1.53)	1.43 (1.19–1.67)	1.49 (1.26–1.73)	1.37 (1.16–1.62)	<0.001	1.46 (1.26–1.70)	1.09 (0.96–1.28)	<0.001
LDL-c, mmol/L	3.26 (2.83–3.75)	3.24 (2.74–3.79)	3.13 (2.66–3.62)	2.99 (2.53–3.58)	3.16 (2.67–3.68)	0.001	3.10 (2.62–3.64)	3.30 (2.79–3.76)	0.002
FPG, mmol/L	4.88 (4.37–5.28)	4.79 (4.36–5.32)	4.74 (4.27–5.19)	4.55 (4.15–5.01)	4.70 (4.28–5.20)	<0.001	4.61 (4.21–5.01)	5.20 (4.53–5.95)	< 0.001
CREA, mmol/L	86.90 (79.20–99.60)	76.55 (64.60–91.50)	70.95 (61.80–85.40)	70.40 (61.80–82.50)	74.50 (63.90–88.30)	<0.001	73.30 (63.00–86.65)	78.00 (65.70–94.60)	<0.001
HOMA_IR	2.36 (1.46–3.64)	1.96 (1.27–3.33)	1.69 (1.10–2.74)	1.43 (0.95–2.20)	1.75 (1.13–2.89)	<0.001	1.52 (0.98–2.36)	2.97 (1.89–4.48)	<0.001
PAD, *n* (%)	21(14.09)	45 (11.08)	51 (10.32)	38 (12.96)	155 (11.54)	0.578	112 (11.11)	43 (12.87)	0.21
Early arterial lesions, *n* (%)	59 (39.60)	133 (32.76)	145 (29.35)	76 (25.94)	413 (30.77)	<0.001	287 (28.47)	126 (37.72)	<0.001
Arterial stiffness, *n* (%)	71 (47.65)	179 (44.09)	166 (33.60)	65 (22.18)	481 (35.84)	<0.001	272 (26.98)	209 (62.57)	<0.001

^*a*^ Values are n (%), or median (IQR).

^*b*^ BMI: body mass index; HOMA-IR: homeostatic model assessment of insulin resistance; TG: triglyceride; TC: total cholesterol; HDL-c: high-density lipoprotein cholesterol; LDL-c: low-density lipoprotein cholesterol; TyG: triglyceride-glucose index; FPG: fasting plasma glucose; WHR: waist-to-hip ratio.

^*c*^ –, MetS status not shown because it duplicates the MetS/non-MetS grouping variable.

### Associations of microbial diversity with HLS and MetS

We then explored the potential role of gut microbiota in HLS and MetS. At the community level, the Shannon diversity index did not significantly differ between the MetS and non-MetS groups. However, the non-MetS group exhibited a higher mean Shannon index than the MetS group (*P* = 0.098, Fig. 1B). Furthermore, the Shannon diversity index was positively associated with HLS categories (*P_trend_* < 0.001, Fig. 1C). Moreover, a significant segregation in beta diversity was observed between the MetS and non-MetS groups (*P* = 0.009, Fig. 1D). A similar significant difference was observed across different HLS categories (*P* = 0.001, Fig. 1E), suggesting a potential link between HLS, gut microbiota alterations, and MetS.

Given that HLS comprises five distinct components, we first associated microbial species with each component to broaden the search for candidate key species. We then visualized the overlap of species across components using a Venn diagram (Fig. 1F). To identify the species most relevant to MetS, we computed Principal Coordinates Analysis axis1 (PCoA1) scores for groups defined by different numbers of overlapping components and assessed their associations with MetS. The group with the largest R^2^ corresponded to species shared by at least three HLS components, which became the focus of our analysis (Fig. 1G). This group contained 20 species, all of which were taken forward for subsequent analyses. These 20 species collectively accounted for 8.88% of the HLS-related variation in MetS (*P* < 0.001, Fig. 1H). Further investigation using logistic and linear regression revealed that out of the 20 species analyzed, five species, *Alistipes putredinis*, *Odoribacter splanchnicus*, *Roseburia hominis*, *Bacteroides ovatus*, and *Prevotella_s CAG_279*, exhibited a significant negative correlation with both MetS and creatinine (Fig. 1I). Overall, our findings indicated that microbial species may serve as intermediaries linking HLS and MetS.

### *Alistipes putredinis*, *Odoribacter splanchnicus,* and *Roseburia hominis* are linked to MetS

To further substantiate the observed associations between the selected microbial species and MetS, we performed bidirectional MR analyses to evaluate potential causal links. Among the five species independently associated with MetS, only *Alistipes putredinis*, *Odoribacter splanchnicus,* and *Roseburia hominis* were found to be significantly associated with MetS in all three MR methods: IVW, simple median, and MR-Egger regression. IVW estimate suggested that *Alistipes putredinis* was negatively associated with MetS (betaIVW = −0.775*,* 95% CI = −1.310 to −0.239, *P*= 0.005*), Odoribacter splanchnicus* (betaIVW *=* −1.770, 95% CI = −3.130 to −0.410, *P* = 0.032*),* and *Roseburia hominis* (betaIVW = −1.555, 95% CI = −2.980 to −0.131, *P* = 0.032. Fig. S2A). Importantly, among the abovementioned associations, the directions of the effect values estimated by IVW were consistent with simple median and MR-Egger regression methods, and reverse MR analysis demonstrated that there were no significant associations of MetS on all three species in any MR method (Fig. S2B through G). On the contrary, the IVW estimate provided no evidence of associations of *Bacteroides ovatus* and *Prevotella_s CAG_279* with MetS (Fig. S3A through D)*.* Additionally, for the two associations observed, the F-statistics of the instrumental variables (IVs) were both greater than 10, eliminating the bias of weak IVs. Moreover, leave-one-out analysis further suggested that the links between *Roseburia hominis*, *Odoribacter splanchnicus*, *Alistipes putredinis,* and MetS were not driven by any single SNP (Fig. S4).

To ensure the generalizability and reliability of our results, we validated them in an external cohort. These three species were found to be lower in individuals with MetS in an independent cohort from China (20) (Fig. S2H). Although there were some fundamental differences in information among these groups, the results are similar, confirming the reliability of the findings (Table S2). Moreover, we tested Shannon index and the three species in two additional MetS definitions, CDS and IDF (Fig. S5). The trends were concordant with those under NCEP-ATP III, with non-MetS groups showing significantly higher Shannon index and higher abundances of the three species. Baseline characteristics of CDS and IDF are provided in Table S3 and S4 We also observed that *Roseburia hominis*, *Odoribacter splanchnicus*, and *Alistipes putredinis* were negatively associated with HOMAIR, TG, and blood pressure. They were also negatively associated with ALT, AST, and baPWV (Fig. S2I). Taken together, these findings demonstrate that *Alistipes putredinis*, *Odoribacter splanchnicus*, and *Roseburia hominis* are important microbial species that are positively associated with HLS and negatively associated with MetS.

### Associations of metabolic capacities of gut microbiota with HLS and MetS

**Fig 2:**
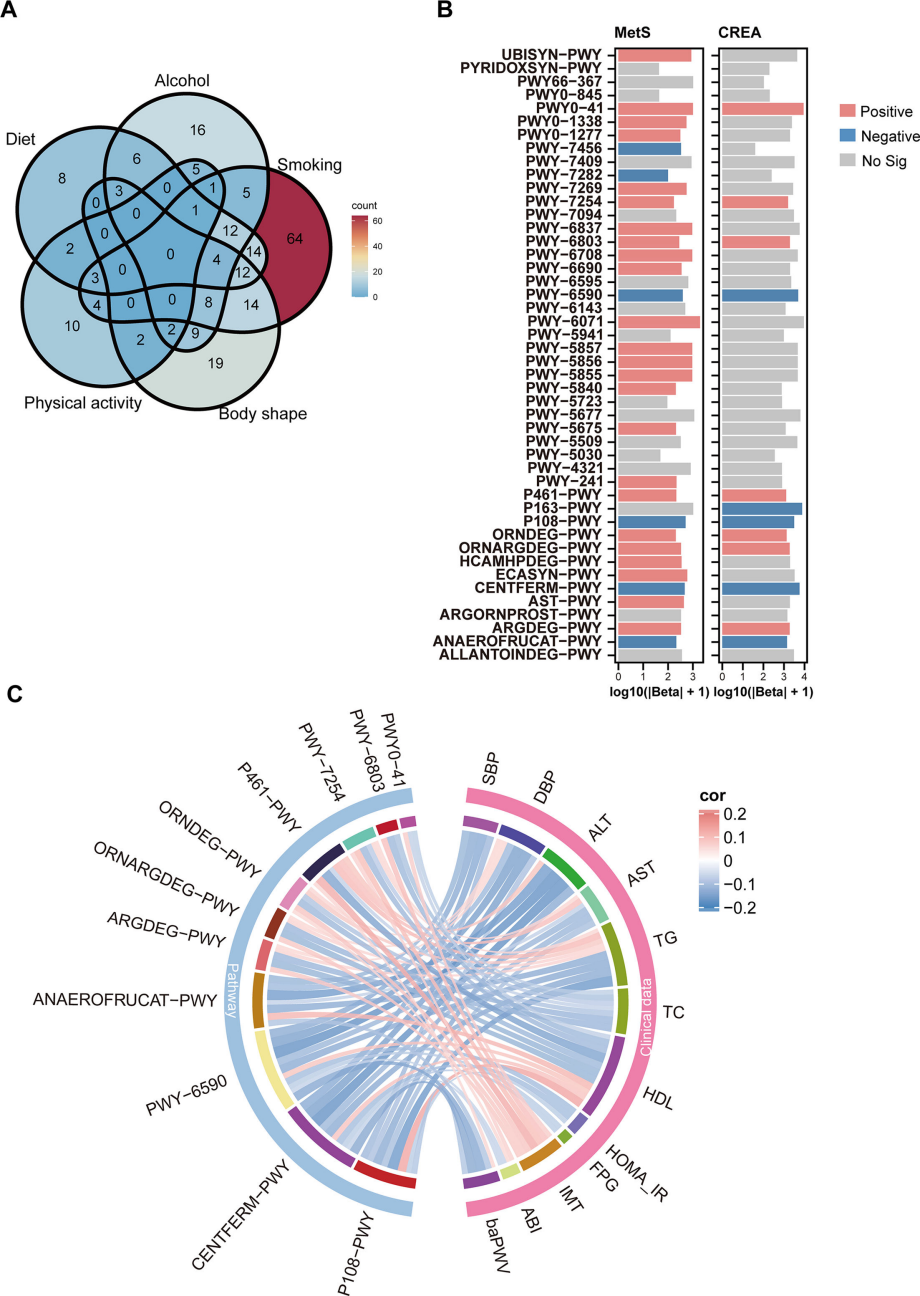
HLS-related microbial functions are closely associated with MetS. (**A**)The Venn diagram illustrates the overlap of microbial functions that are significantly associated with five lifestyle factors: body shape, diet, physical activity, smoking, and alcohol. (**B**)The independent association of 46 microbial functions with MetS and CREA adjusted for age, sex, education level, medication use, and family medical history. Red, blue, and gray indicate positive, negative, and insignificant correlations with MetS (*P*_adj_ values were determined by logistic regression, with *P*_adj_ < 0.05), respectively. (**C**) The chord diagram shows the association between negative MetS-related microbial functions and clinical parameters (positive in red and negative in blue) (*P*_adj_ values were determined by Spearman’s correlation, with *P*_adj_ < 0.05).

To elucidate the mechanism underlying the association of the gut microbiota with MetS and HLS, we investigated the functional profiles of microbial MetaCyc pathways across all study participants. We selected pathways significantly linked to each of the five HLS components and visualized their differences and overlaps using a Venn diagram (Fig. 2A). We identified 46 pathways that were significantly associated with at least three HLS components for further analysis. Among these, 11 pathways were found to be significantly associated with both MetS and CREA (Fig. 2B). Four pathways were significantly negatively correlated with both MetS and CREA. PWY-6590 and CENTFERM-PWY are responsible for generating butyrate, and P108-PWY is involved in the production of propionate, which activates GPR41/GPR43 on intestinal epithelial cells to enhance insulin sensitivity, attenuate inflammation, and upregulate the expression of tight-junction proteins (36). Additionally, lactate from the ANAEROFRUCAT-PWY homolactic fermentation pathway serves as an energy substrate for enterocytes, fosters commensal probiotic growth, and modulates hepatic glucose and lipid metabolism (37). In contrast, the arginine and ornithine catabolic superpathways (ORNARGDEG-PWY, ARGDEG-PWY) substantially deplete host arginine reserves, impair endothelial nitric oxide synthesis, and compromise endothelial function, leading to vascular relaxation deficits and an elevated risk of metabolic disease (38). These pathways were also closely linked to established MetS risk factors such as blood pressure, BMI, TG, HOMA-IR, FPG, baPWV, IMT, and ABI (Fig. 2C).

### Associations of metabolites of gut microbiota with HLS and MetS

**Fig 3:**
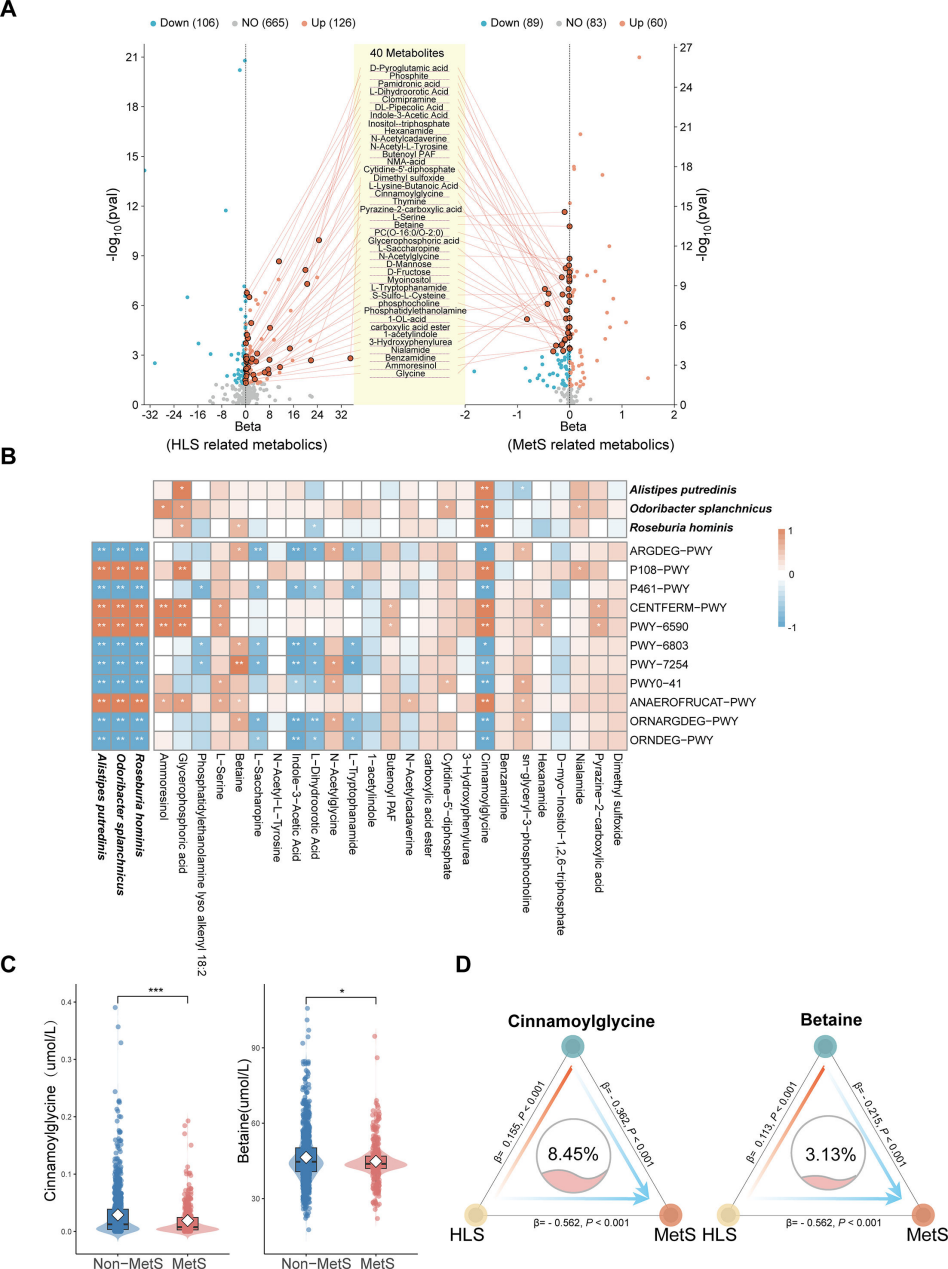
Microbial metabolites mediate the beneficial effect of HLS on MetS. Correlation between selected microbial features and plasma metabolites. (**A**) Double volcano plot illustrates the overlapping metabolites that are significantly upregulated in HLS and significantly negatively correlated with MetS. (**B**) Heatmap of Spearman’s correlation coefficients between selected microbial metabolites, selected functional pathways, and species. All *P* values were adjusted for multiple comparisons. * *P*_adj_ < 0.05, **P_adj_ < 0.01*.* (**C**) Distribution of betaine and cinnamoylglycine in subjects with MetS or non-MetS by targeted metabolomics analysis. * *P* < 0.05, **P < 0.01, ***P < 0.001. (**D**) Mediation linkages among HLS, betaine, and cinnamoylglycine targeted metabolomics, and MetS, adjusted for age, sex, education level, medication use, and family medical history.

Metabolites, the small functional molecules produced through microbial metabolism, play a critical role in the mechanism of gut microbiota. To pinpoint the metabolites that link HLS to MetS, we employed a combination of metagenomics and plasma metabolomics to explore how healthy behaviors correlate with MetS through the gut microbiota. In total, 897 metabolites were measured, out of which 126 showed significant positive correlations with HLS (Fig. 3A), and 25 of these were both negatively associated with MetS and creatinine (Fig. 3A and Fig. S6).

Cinnamoylglycine and betaine were significantly positively correlated with HLS and negatively correlated with MetS. The abundance of butyrate-producing bacteria such as *Roseburia hominis*, *Odoribacter splanchnicus*, and *Alistipes putredinis* was positively correlated with cinnamoylglycine and the pyruvate metabolic pathway (Fig. 3B). Notably, glucose-6-phosphate isomerase, a key enzyme involved in amino sugar and nucleotide sugar metabolism, can be annotated in the genomes of *Alistipes putredinis* and *Roseburia hominis* through protein sequence BLAST analysis (Table S5). Similarly, as a consecutive enzyme in glycolysis and gluconeogenesis, class I fructose-bisphosphate aldolase has been identified in *Odoribacter splanchnicus* (Fig. S7). Enzymes essential for glutamate production are activated by the high abundance of *Alistipes putredinis*, *Odoribacter splanchnicus*, and *Roseburia hominis* in non-MetS subjects, leading to significant consumption of β-D-fructofuranose 6-phosphate and D-fructose 1,6-bisphosphate. These metabolites, key precursors in the ANAEROFRUCAT-PWY pathway, increase hormone fermentation and increase intermediate metabolites like phosphoenolpyruvate, a precursor for betaine and cinnamoylglycine synthesis. Additionally, all three species possess the glyA and aroC genes (Fig. S7). The glyA gene encodes glycine hydroxymethyltransferase, which facilitates glycine production (39), whereas the aroC gene encodes chorismate synthase, which promotes the synthesis of phenylalanine (40). Both processes can further enhance the production of betaine and cinnamoylglycine.

The level of betaine was strongly negatively correlated with MetS and positively correlated with HLS and butyrate-producing bacteria. As a metabolite of glycine and serine, betaine serves as a methyl donor in methylation, a vital process in humans, including the methionine cycle (41). Reduced levels of L-serine, glycine, and L-sarcosine in MetS subjects likely hindered betaine production (Fig. S7). Similarly, cinnamoylglycine and betaine, both gut microbiota-derived metabolites, were positively associated with HLS and negatively with MetS. Cinnamoylglycine biosynthesis was linked to butyrate-producing bacteria and the pyruvate pathway, both enhanced by HLS, while betaine production depended on increased levels of L-serine, glycine, and L-sarcosine (42).

Furthermore, targeted metabolomics analysis revealed that the median levels of betaine and cinnamoylglycine were 44.7 μmol/L and 0.125 μmol/L (Fig. 3C), closely mirroring those reported by Schwahn’s study (43), respectively, in individuals with non-MetS, which were considerably higher than the levels observed in subjects with MetS. Additionally, a mediation model specified HLS as the independent variable, MetS as the dependent variable, and betaine and cinnamoylglycine as mediating variables. The mediation model revealed that cinnamoylglycine had a significant indirect effect of HLS on MetS (proportion mediated = 8.45%, *P* = 0.001, Fig. 3D). The effect of betaine also remained significant, with an indirect effect (proportion mediated = 3.13%, *P* = 0.001, Fig. 3D).

### ROC curves of cinnamoylglycine and betaine in relation to arterial stiffness, early arterial lesions, and PAD

**Fig 4:**
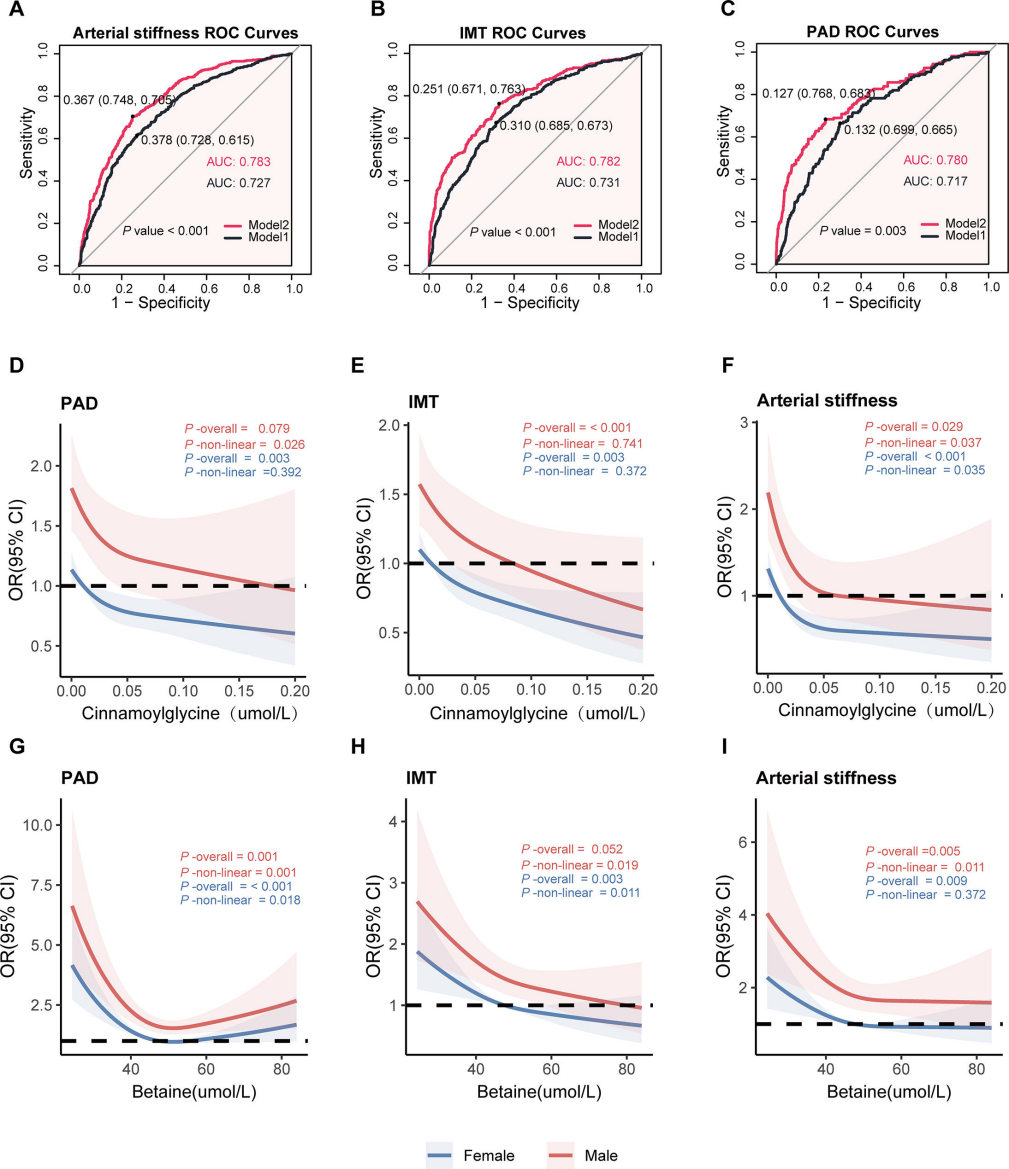
Predictive performance of two models and sex-specific dose-response associations between circulating metabolites and arterial stiffness, IMT, and PAD. (**A–C**) ROC curves for Model 1 (black) versus Model 2 (red) in predicting IMT, arterial stiffness, and PAD. Model 1 included central obesity, TG, HDL-C, blood pressure, and fasting glucose; Model 2 comprised all of these variables plus serum cinnamoylglycine and betaine. (**D–F**) Restricted cubic spline curves relating cinnamoylglycine (μmol/L) to adjusted odds ratios (ORs; dashed line at OR = 1) for PAD (**D**), IMT (**E**), and arterial stiffness (**F**). Red and blue curves denote males and females, respectively; shaded bands indicate 95% confidence intervals. Overall and sex-specific nonlinearity *P*-values are reported in each panel (**G–I**). Analogous spline analyses for betaine concentration (μmol/L) versus PAD (**G**), early arterial lesions (IMT) (**H**), and arterial stiffness (**I**), with color-coding, reference line, and *P*-values as in D–F, respectively.

A refined prediction model was developed by integrating the five MetS components with circulating levels of cinnamoylglycine and betaine. Model 1 included central obesity, TG, HDL-C, blood pressure, and fasting glucose; Model 2 included all of these variables plus serum cinnamoylglycine and betaine. ROC analyses revealed that Model 2 was significantly superior to the traditional risk factor model in terms of discriminative power for arterial stiffness (AUC 0.783 vs 0.727, *P* < 0.001), early arterial lesions (IMT: AUC 0.782 vs 0.731, *P* < 0.001), and PAD (AUC 0.780 vs 0.717, *P* = 0.003) compared with the traditional risk-factor model (Fig. 4A through C).

### RCS analysis investigating the relationships between cinnamoylglycine and betaine and arterial stiffness, early arterial lesions, and PAD

We used RCS to flexibly model and visualize the sex-specific associations between serum cinnamoylglycine and betaine and three vascular outcomes (PAD, IMT, and arterial stiffness), adjusted for age, education, and smoking habits, medication use, and family medical history in the model. For cinnamoylglycine, there was a linear relationship with IMT in both women (*P*-overall = 0.003; *P*-nonlinear = 0.372) and men (*P*-overall < 0.001; *P*-nonlinear = 0.741). Nonlinear associations were observed with arterial stiffness in women (*P*-overall < 0.001; *P*-nonlinear = 0.035) and men (*P*-overall = 0.029; *P*-nonlinear = 0.037), as well as with PAD in women (*P*-overall = 0.003; *P*-nonlinear = 0.392) and men (*P*-overall = 0.079; *P*-nonlinear = 0.026). Similarly, betaine exhibited nonlinear associations with PAD in women (*P*-overall < 0.001; *P*-nonlinear = 0.018) and men (*P*-overall = 0.001; *P*-nonlinear = 0.001) and with IMT in women (*P*-overall = 0.003; *P*-nonlinear = 0.011) and men (*P*-overall = 0.052; *P*-nonlinear = 0.019). Its association with arterial stiffness was linear in women (*P*-overall = 0.009; *P*-nonlinear = 0.372) but nonlinear in men (*P*-overall = 0.005; *P-nonlinear*P-nonlinear = 0.011) (Fig. 4D through I).

### Downstream targets of cinnamoylglycine and betaine

To identify the molecular targets through which the selected metabolites may influence MetS, we performed *in silico* target profiling of human proteins, including transporters and receptors. Overall, 89% of the downstream targets of cinnamoylglycine and betaine have been implicated in the development of MetS, among which 41 targets were confirmed using overlapping both databases (Fig. S8A). For instance, FABP4 is a lipid chaperone primarily expressed in adipocytes and macrophages. It plays a critical role in lipid metabolism and inflammatory responses. Elevated levels of FABP4 are associated with insulin resistance, obesity, and an increased risk of developing MetS and CVDs (44). Similarly, ABCG2 is involved in drug and lipid transport, linking cholesterol metabolism and drug resistance to MetS and vascular health (45). Gene Ontology (GO) enrichment analysis suggested that the downstream targets of these metabolites were primarily involved in metabolism and response to several signaling pathways (Fig. S8B). Notably, several GO terms related to the cellular response to lipid or fatty acid metabolic process signaling were identified (Fig. S8C), further suggesting that key species may promote the development of insulin resistance via the insulin receptor signaling pathway.

## DISCUSSION

We aimed to investigate the association between HLS and the gut microbiome, as well as its relationship with MetS and vascular health, to further elucidate the mechanisms by which the microbiome functions in this context. In a cohort of 1,342 community individuals, we performed deep characterization via metagenomic sequencing, metabolomics, and host genotyping. We found that *Roseburia hominis*, *Odoribacter splanchnicus*, and *Alistipes putredinis* were correlated with HLS, MetS, and the inflammatory marker serum CREA. Through integrative multiomics targeted detection, we further identified cinnamoylglycine and betaine as key molecular mediators linking MetS and vascular health. While our analyses defined MetS using the NCEP-ATP III criteria to ensure international comparability, we also reclassified MetS according to the CDS 2017 criteria, which are widely used in China, and performed a sensitivity analysis. The CDS-based results retained the direction and statistical significance of the principal gut microbiota associations, underscoring the robustness of our findings.

Emerging research indicates that the gut microbiota can influence host lipid metabolism, insulin resistance, and inflammatory responses. An exercise intervention study in Asian prediabetic patients revealed significant interindividual differences in improvements in insulin resistance, depending on distinct gut microbiota compositions, highlighting the regulatory role of the microbiota in lifestyle interventions (46). Similarly, a recent study of 2,309 individuals from Europe identified close associations between 32 microbial families and genera and various lipoprotein particles (47). The Mind-Body Study, part of the Nurses’ Health Study II, also found strong associations among gut microbiota, physical activity, and body weight (48). However, these studies mainly involved populations of European descent and did not adequately control for confounding by comorbidities. Considering the significant impacts of genetic background, diet, lifestyle, and geography on the microbiota, substantial differences exist in core microbial features and their associations with host metabolism among different ethnic groups. In this context, we were the first to conduct a large-scale investigation in 1,342 Chinese individuals using metagenomic sequencing to systematically elucidate the relationships among healthy lifestyle, gut microbiota, and MetS. With the advantage of metagenomics, we found that the abundances of *Alistipes putredinis*, *Odoribacter splanchnicus*, and *Roseburia hominis* remained significantly positively associated with HLS but negatively associated with MetS and serum CREA. MR analysis further confirmed associations between these three bacteria and MetS.

Both *Alistipes putredinis* and *Odoribacter splanchnicus* belong to the Bacteroidetes phylum, and previous studies have shown that *Odoribacter splanchnicus* can produce short-chain fatty acids (SCFAs) (49, 50), while *Alistipes putredinis* also positively correlates with SCFA levels (51), which have anti-inflammatory effects that help reduce low-grade inflammation (52). In a cohort of 2,262 Chinese individuals, *O. splanchnicus* and *R. hominis* were identified as obesity-related biomarkers (53). Research from the U.S. Health Professionals Follow-up Study and Nurses’ Health Study II demonstrated that these bacteria mediate the relationship between physical activity and body weight (48). Compared to healthy and obese controls, patients with non-alcoholic steatohepatitis exhibited reduced abundance of *Alistipes putredinis* (47). *In vitro* studies of *Roseburia hominis* have demonstrated β-glucosidase activity (54), suggesting its involvement in xenobiotic metabolism and the bioavailability of dietary bioactive compounds. These findings are consistent with our results, which show that these three bacteria were negatively correlated with TC and TG and significantly negatively correlated with atherosclerosis.

The lack of microbiome functional profiling and comprehensive metabolite detection is a notable limitation of previous large-scale population studies (55). Unlike earlier investigations that relied mainly on 16S rRNA gene sequencing and therefore had limited functional resolution, our metagenomic analysis revealed that the reductive homolactic fermentation (ANAEROFRUCAT-PWY) capacity of the microbiota was significantly suppressed in MetS and positively correlated with the three identified species. Among physically active individuals, the ANAEROFRUCAT-PWY and related species were positively associated with training volume and muscle mass, suggesting that exercise and greater lean mass may be linked to activation of this pathway (56). Similar to our observation, a population-based study showed that the ANAEROFRUCAT-PWY was inversely associated with unhealthy dietary patterns and alcohol intake (57). The same study also reported a positive association between ANAEROFRUCAT-PWY and *Lachnospiraceae bacterium 8_1_57FAA*, a species implicated in blood pressure regulation through inflammatory pathways. In a randomized controlled trial, supplementation with *Lacticaseibacillus casei LC2W* enhanced the lactate fermentation pathway, attenuated intestinal inflammation, and promoted glucose and lipid catabolic pathways, thereby ameliorating features of MetS (58). Consistent with the above studies, our work not only considered both species composition and functional profiles of the gut microbiome but also integrated widely targeted and targeted metabolomics to identify microbial metabolites, thereby providing a more robust foundation for subsequent research.

Multi-omics integrative analysis indicated that, consistent with the accumulation of fermentation intermediates, cinnamoylglycine is a glycine conjugate of cinnamic acid and that it is known to be produced by gut microbes because it is abundant in the serum of conventional mice but present in minimal concentrations in the serum of germfree mice (59, 60). Consistently, previous work shows that circulating cinnamoylglycine is inversely related to insulin resistance, adiposity, and TG (60). Cinnamoylglycine is also a recognized marker of peroxisome-proliferator-activated receptor-α (PPAR-α) activity. PPAR-α signaling inhibits atherogenic lipid production and helps preserve myocardial lipid homeostasis, offering a mechanistic link between cinnamoylglycine and cardiometabolic protection (61). Whereas cinnamoylglycine emerges as a downstream effector regulating that lipid handling, insulin signaling, and immune tone, betaine enters the metabolic cascade one step earlier. Enhanced homolactic fermentation flux in the gut microbiome elevates succinate levels, and this rise in succinate amplifies the cellular pools of L-serine and L-glycine—two direct precursors of betaine. The newly synthesized betaine then remethylates homocysteine to methionine via BHMT, thereby lowering circulating homocysteine and attenuating endothelial injury and atherogenesis (62). Experimental study further shows that betaine activates AMPK, suppresses the lipogenic regulator SREBP-1c and fatty-acid synthase (63), and reduces hepatic and plasma lipid accumulation. Concurrently, it enhances insulin signaling (phosphorylation of IRS-1 and Akt), diminishes NF-κB-driven expression of TNF-α and IL-6, limits reactive-oxygen production, and upregulates eNOS (64, 65).

MetS is characterized by low-grade chronic inflammation, which significantly affects vascular health (66). In our study, the microbes and microbial functions most closely linked to MetS and CREA were also strongly associated with vascular indices—PAD, IMT, and arterial stiffness. Current research shows that combining clinical indicators with biomarkers can improve predictive power (67). Our study established a model integrating selected metabolites with MetS components, exhibiting superior predictive performance compared to using MetS components alone. ROC analysis demonstrated that this model presented high sensitivity and specificity for outcomes including arterial stiffness, early arterial lesions, and PAD. RCS analysis stratified by sex revealed significant negative correlations between cinnamoylglycine and betaine with arterial stiffness, early arterial lesions, and PAD, with most associations exhibiting significant nonlinearity. This aligns with the results of previous studies indicating that metabolite dose-response relationships in humans are largely nonlinear (68) potentially due to secondary metabolite effects, for instance, betaine’s secondary metabolite trimethylamine N-oxide being harmful on cardiovascular function. Females exhibited lower cardiovascular risk than males, consistent with prior reports (69).

### Study limitations

Several limitations should be acknowledged. First, the study was conducted at a single center in southern China, and nearly all participants were of Chinese ancestry. Consequently, the results should be generalized to other ethnic groups with caution; additional studies in diverse populations and settings are warranted. Second, the cross-sectional design allows only for correlation, not causation. Mechanistic work in preclinical models and longitudinal cohorts is needed to clarify causal pathways. Third, we did not perform experimental validation, which is essential for confirming the robustness of our findings. Future research will therefore include external validation in multiple centers and experimental confirmation in animal models. Finally, although we identified associations with SCFA-producing species, canonical SCFAs cannot be captured by our untargeted liquid chromatography-mass spectrometry method and, therefore, could not be directly associated in this data set. This reflects a known analytical constraint: metabolomics and lipidomics target chemically distinct analytes and often require different extraction and ionization strategies; volatile, low-molecular-weight organic acids such as SCFAs typically necessitate targeted assays for reliable quantitation (70). In future work, we will include targeted SCFA profiling and integrate these data with microbial species and host lipidome in a unified analysis. Such an integrated design provides complementary coverage and strengthens causal inference across interconnected pathways, improving mechanistic interpretation of microbiome-metabolite-host links.

### Conclusion

Our study revealed significant links among lifestyle factors, the gut microbiota, microbially derived metabolites, MetS, and vascular health. In particular, we identified cinnamoylglycine and betaine as key gut microbiota-mediated metabolites that strongly inversely associated with both MetS and vascular disease. These findings enhance our understanding of how daily habits, the gut microbiome, and host metabolism interact and provide scientific support for microbiome-based strategies to prevent and manage MetS and vascular disorders.

## Data Availability

The raw sequence data reported in this paper have been deposited in the Genome Sequence Archive (Genomics, Proteomics & Bioinformatics 2025) in National Genomics Data Center (Nucleic Acids Res 2025), China National Center for Bioinformation / Beijing Institute of Genomics, Chinese Academy of Sciences (GSA: CRA033854), and are publicly accessible at https://ngdc.cncb.ac.cn/gsa. Other data that support the findings of this study are available from the corresponding authors upon reasonable request. The STORMS reporting checklist has been archived in Figshare (DOI: 10.6084/m9.figshare.30570665).

## SUPPLEMENTAL MATERIAL

Supplemental Material msystems.01433-25-s0001.docx Supplemental methods, Figures S1 to S8, and Tables S1 to S5.

Supplemental Tables msystems.01433-25-s0002.xlsx Tables S6 to S8.

ASM does not own the copyrights to Supplemental Material that may be linked to, or accessed through, an article. The authors have granted ASM a non-exclusive, world-wide license to publish the Supplemental Material files. Please contact the corresponding author directly for reuse.
