# Supplementary material for: Microbial metabolites associated with healthy lifestyles in relation to metabolic syndrome and vascular health: a cross-sectional study
Source: mSystems. 2025 Dec 23;11(2):e01433-25. doi: 10.1128/msystems.01433-25 (PMC12911354; doi:10.1128/msystems.01433-25)
Supplement: Supplemental Material — Supplemental methods, Figures S1 to S8, and Tables S1 to S5. [file msystems.01433-25-s0001.docx]

**SUPPLEMENTAL MATERIAL TO**

**Microbial metabolites associated with healthy lifestyles in relation to metabolic syndrome and vascular health: a cross-sectional study**

Zhuoyu Zhang^1^, Bingqi Ye^2^, Jialin He^1^, Li Xiang^2^, Siqi Li^1^, Jiaqi Zhao^1^, Wanlan Chen^1^, Qi Zhang^1^, Wanying Zhao^1^, Jialu Yang^1^ , Yi Li^1^, Jingmeng Ju^1^, Yan Liu^1*^, Min Xia^1*^

^1^Guangdong Provincial Key Laboratory of Food, Nutrition and Health, and Department of Nutrition, School of Public Health, Sun Yat-sen University

^2^Guangdong Provincial Key Laboratory of Food, Nutrition and Health, and Department of Statistics and Epidemiology, School of Public Health, Sun Yat-sen University

**Corresponding authors:** Dr. Yan Liu, Department of Nutrition, School of Public Health, Sun Yat-sen University, P.R. China. E-mail: [liuyan215@mail.sysu.edu.cn](mailto:liuyan215@mail.sysu.edu.cn). OR Prof. Min Xia, Department of Nutrition, School of Public Health, Sun Yat-sen University, P.R. China. E-mail: [xiamin@mail.sysu.edu.cn](mailto:xiamin@mail.sysu.edu.cn).

Table of contents

Supplementary Methods 3

Supplementary Results 17

Supplemental Figure 1. Flow chart for study participant recruitment and data analysis.........................................................................................................................17

Supplemental Figure 2 HLS-related species are linked to MetS. 18

Supplemental Figure 3. The relative abundance of species. 20

Supplemental Figure 4. Leave-one-out plots for the causal association between Roseburia hominis, Odoribacter splanchnicus, Alistipes putredinis, Prevotella sp_CAG_279, Bacteroides ovatus and MetS. 21

Supplemental Figure 5. Differences in gut microbial diversity and species between MetS and Non‑MetS groups classified by CDS and IDF criteria. 23

Supplemental Figure 6. The independent association of metabolites with creatinine(CREA). 25

Supplemental Figure 7. Microbial metabolites serve as effectors to MetS. 26

Supplemental Figure 8. Predicted targets and enriched pathways of key metabolites and MetS. 28

Supplemental Table 1. MetaCyc ID and the corresponding names of each pathway 30

Supplemental Table 2. Baseline characteristics of the study participants in discovery and validation cohort 32

Supplemental Table 3. Baseline characteristics according to the CDS. 33

Supplemental Table 4 Baseline characteristics according to the IDF. 35

Supplemental Table 5 Alignment results for bacterial key enzymes related to metabolites. 37

References 38

# Supplementary Methods

**Collection of metadata**

Information on demographic characteristics, medical history, medication use, lifestyles, and prevalence of comorbidities were collected with structured questionnaires by trained staff. Blood pressure was measured twice on the right upper arm in the sitting position after at least 5 minutes of rest using a validated digital automatic analyzer (Omron HEM-7136) and the average level was used. Weight was measured with Tanita bioelectrical impedance device (TANITA-BC-601; Tanita Corp)[1]. Blood samples after an overnight fast for 10-12h were collected and analyzed immediately at local laboratory. Measurements of glucose, Total Cholesterol (TC), triglycerides (TG), High‑Density Lipoprotein Cholesterol (HDL-c) and Low‑Density Lipoprotein Cholesterol (LDL-c) were assessed by enzymatic methods on a Microplate Reader (Mindray BS800M; Mindray, Shenzhen, China). Insulin levels were measured with commercial ELISA kits (Mercodia, Uppsala, Sweden). Homeostatic model assessment for insulin resistance (HOMA-IR) was then calculated as fasting insulin (μU/L) × fasting glucose (mmol/L)/22[2].

**Definition of healthy lifestyles**

**Cigarette smoking:** Smoking status was defined as self-reported tobacco use, and was classified into three groups of daily (more than 1 cigarette per day or 7 cigarettes a week for the past half a year), former (quit smoking for more than half a year) and never smokers. Not smoking or quitting smoking for reasons other than illness were classified as healthy behavior.

**Drinking:** Drinking was categorized into four groups of heavy, moderate, occasional and never drinkers. Past and current alcohol drinking patterns were self-reported. Participants were classified as heavy drinker (some alcohol use in most weeks in the past year and weekly alcohol use >140 g in men or 70 g in women ), moderate drinker (some alcohol use in most weeks in the past year and weekly alcohol use <140 g in men or 70 g in women ), never drinkers (no alcohol use in the past year and never drank in most weeks), occasional drinkers (occasional alcohol use in the past year but never drank in most weeks)[3]. No heavy drinking was deemed as healthy.

**Physical activity:** The International Physical Activity Questionnaire Short Form (IPAQ-SF)[4] was used to collect the information of physical activity. Engaging in at least 150 to 300 minutes of moderate-intensity aerobic physical activity each week, or 75 to 150 minutes of vigorous-intensity aerobic physical activity, is defined as a healthy behavior [5].

**Diet:** Information on diet was collected using a validated Food Frequency Questionnaire. Participants were asked how often they consumed various food items, including vegetables, fruits, nuts, red meat, white meat, eggs, fish, milk and dairy products—eight major food groups traditionally included in the Chinese diet. The intake frequency of each food group was measured on a 5-point scale: ‘almost every day’, ‘at least once per week’, ‘at least once per month’, ‘occasionally’, and ‘rarely or never’. If the response for a food group was ‘almost every day’ or ‘at least once per week’, one point was given; otherwise, no point was given. Thus, the diet diversity score ranged from 0 to 8, with a higher score indicating greater dietary diversity. A dietary diversity score of 6 points or above was considered healthy[6].

**Body shape:** A healthy body composition is defined as having a BMI between 18.5 and 24.9 kg/m², in addition to no abdominal obesity, which is determined by a waist-to-hip ratio (WHR) of ≥ 0.9 for men and ≥ 0.85 for women. Both criteria must be met to be classified as having a healthy body shape[7,8].

**Assessment of MetS**

**National Cholesterol Education Programme-Adult Treatment Panel III criteria**： According to the National Cholesterol Education Programme-Adult Treatment Panel III (NCEP-ATP III), MetS was defined as having three or more metabolic disorders: (1) central obesity, defined as waist circumference≥90 cm in males, or ≥80 cm in females; (2) elevated blood pressure, defined as systolic blood pressure (SBP) ≥130 mmHg, or diastolic blood pressure (DBP) ≥85 mmHg, or treatment of previously diagnosed hypertension; (3) elevated TG, defined as having a fasting TG ≥1.7 mmol/L; (4) reduced HDL-c, defined as having HDL-c <1.03 mmol/L in males, or <1.30 mmol/L in females; and(5) elevated fasting plasma glucose (FPG), defined as having an FPG ≥5.6 mmol/L, or use of any hypoglycemic drugs[9].

**International Diabetes Federation** **criteria**: According to the International Diabetes Federation (IDF), MetS is defined as central obesity (defined as waist circumference ≥90 cm in men or ≥80 cm in women for Chinese populations) plus any two of the following four factors: (1) elevated triglycerides (TG), defined as having a fasting TG ≥1.70 mmol/L (150 mg/dL) or being on specific treatment for this lipid abnormality; (2) reduced high-density lipoprotein cholesterol (HDL-C), defined as having HDL-C <1.04 mmol/L (40 mg/dL) in males or <1.29 mmol/L (50 mg/dL) in females, or being on specific treatment for this lipid abnormality; (3) elevated blood pressure, defined as SBP ≥130 mmHg or DBP ≥85 mmHg, or treatment of previously diagnosed hypertension; and (4) elevated fasting plasma glucose (FPG), defined as having an FPG ≥5.6 mmol/L (100 mg/dL), or previously diagnosed type 2 diabetes[10].

**Chinese Diabetes Society (CDS) criteria**: According to the Chinese Diabetes Society (CDS), MetS is defined as having three or more of the following metabolic disorders: (1) central obesity, defined as waist circumference ≥90 cm in males or ≥85 cm in females; (2) TG, defined as having a fasting TG ≥1.70 mmol/L (150 mg/dL); (3) HDL-C , defined as having HDL-C <1.04 mmol/L (40 mg/dL) for both males and females; (4) elevated blood pressure, SBP ≥130 mmHg or DBP ≥85 mmHg, or having a history of previously diagnosed hypertension; and (5) elevated glucose, defined as having a FPG ≥6.1 mmol/L, or a 2-hour plasma glucose ≥7.8 mmol/L, or having been previously diagnosed with diabetes[10].

**Power analysis**

Power and sample-size calculations were performed with the R package micropower, which implements the distance-matrix simulation framework of Kelly et al(9). Using unweighted UniFrac distances, we matched the observed within-group distribution in a 50-sample pilot set (mean μ = 0.84, SD σ = 0.048) and simulated 1 000 bootstrap PERMANOVA runs for the actual, design (MetS n = 334; non-MetS n = 1008). The study achieves ≥80 % power (α = 0.05, two-sided) to detect very small between-group effects (ω² ≥ 0.006) and >99 % power for our pilot effect estimate (ω² ≈ 0.04); therefore, the current sample size (N = 1342) is sufficient and no further recruitment was required.

**Quality control, taxonomy profiling and functional annotation for metagenomics data**

The trimming and filtering of adaptors in metagenomics data were performed by fastp[11]. Human contaminants were further removed by mapping against the host genome reference (hg38) with bowtie2 (v2.4.1, default parameters; coverage >80% of read)[12]. On average, 55.3 million high-quality reads per sample were generated for subsequent analyses. Taxonomic profiling was performed using MetaPhlAn3 (v3.0.13) with default parameters. Functional profiles for the metagenomes were obtained using the HUMAnN3 pipeline (v3.0.0, uniref 90 diamond database), with its built-in database (MetaCyc database, https://MetaCyc.org)to identify metabolic capacities, also known as metabolic pathways, which refer to the functional abilities of the gut microbiota to metabolize substrates and produce various metabolites[13]. All taxa and pathway data were reported as relative abundances (total sum scaling normalization) in each sample for subsequent analysis.

Vegan package was used to calculate alpha diversity. The association between each lifestyle factors and microbial diversity (Shannon index) was evaluated by linear regression. The association between MetS and Shannon index was evaluated by Wilcoxon Signed Rank Test. The Permutational multivariate analysis of variance (PERMANOVA) with the Adonis function was used to estimate community diversity at the species level. To evaluate whether microbial diversity mediated the impact of HLS on MetS, we applied the mediation analysis using mediation function from the R package “Mediation”. The mediation model was adjusted for age, sex, education level, medication use, and family medical history, and the results were confirmed by the simulation exercise bootstrapped 100 times.

After filtering out low-prevalence microbiome features, defined as species or metabolic functions with a detection rate less than 5% in all subjects, microbial features were log-transformed and subjected to further analyses.

**Metabolomics profiling in plasma samples**

For plasma metabolic proﬁling, an integrated method for large-scale detection, identiﬁcation and quantiﬁcation of widely targeted metabolites was employed as previously described with minor revision[14] by Metware (Wuhan, China). A total of 50 μL plasma samples were thawed on ice, vortex for 10 sec, followed by the addition of 300 μL of pure methanol with internal standards, including L-2-chlorophenylalanine, [^2^H_3_]-L-carnitine HCl, 4-fluoro-L-α-phenylglycine, L-phenylalanine (2-^13^C, 99%), [^2^H_5_]-hippuric acid, [^2^H_5_]-kynurenic acid, [^2^H_5_]-phenoxy acetic acid. The mixture of plasma and internal standards was vortexed for 3 min, and the supernatants were recovered by centrifugation at 12,000 rpm for 10 min at 4°C. Then, 10 μL of the supernatant taken from each sample was pooled together to create a “mixed sample”, followed by a full scan mode with QTRAP^®^ 6500+ system to acquire a high-resolution data including RT, Q_1_, and Q_3_. Peak annotation of the mixed sample was performed with the in-house database of Metware Co., Ltd (Wuhan, China), followed by a transfer of the ion pair information to the triple quadrupole (QQQ) instrument. Finally, individual plasma samples were analyzed by QQQ in multiple reaction monitoring (MRM) mode to get a more accurate quantification of metabolites. More details for this process and instrument setup were as follows: chromatographic separation was performed on ACQUITY UPLC HSS T3 C18 (1.8 µm, 2.1 mm×100 mm, Waters) using an UPLC system (Shim-pack UFLC SHI-MADZU CBM A system, https://www.shimadzu.com/; QTRAP^®^ 6500+ System, https://sciex.com/). Mobile phase A was 0.1% formic acid in water, and mobile phase B was 0.1% formic acid in acetonitrile. Mass spectrometry (MS) detection was performed by triple quadrupole-linear ion trap mass spectrometry (QTRAP), equipped with an electrospray ionization (ESI) source. In chromatographic separation 2 µL of sample was used and the flow rate was set at 0.35 mL/min. Full scans with a range of m/z 50-1000 were acquired in LIT and QQQ scans with positive and negative ion modes and controlled by Analyst 1.6.3 software (Sciex). The ESI source parameters were as follows: source temperature 500°C; ion spray voltage (IS) 5500 V (positive), -4500 V (negative); ion source gas I (GSI), gas II (GSII), curtain gas (CUR) was set at 55, 60, and 25.0 psi, respectively; the collision gas (CAD) was high. Instrument tuning and mass calibration were performed with 10 and 100 μmol/L polypropylene glycol solutions in QQQ and LIT modes, respectively. A specific set of MRM transitions were monitored for each period according to the metabolites eluted within this period.

**Statistical analysis of metagenomics and metabolomics data**

***Selection of microbial features independently associated with HLS and MetS***

After filtering out low-prevalence microbiome features (defined as a detection rate less than 5% for species, and 20% for metabolic functions in all subjects), to find accessory genes associated with HLS and MetS, we used a similar linear regression model(GLM) with respect to the presence or absence of the gene (Z; 0 or 1 for absence or presence, respectively) adjusted by species abundance in addition:

***Log(Y) ~ Z + Log(X) + Age + Sex + education level+medication use+ family medical history***

And a permutation-based (1000×) false-discovery rate-corrected *P* value was estimated for multiple comparisons. linear regression analysis was firstly applied to identify species significantly associated with HLS (*P*_adj_ < 0.05). For each HLS component (binary: diet, alcohol, smoking, physical activity, body shape), we tested associations with species, and *P*_adj_ < 0.05 was considered significant. We grouped species by the number of HLS components in which they were significant (intersection count = 5; ≥4; ≥3; ≥2; ≥1). For each group, we summarized participant‑level microbiome variation using principal coordinates analysis (PCoA) on Euclidean distances of CLR‑transformed abundances (Aitchison distance). The first principal coordinate (PCoA1) was extracted for each participant, standardized (z‑score), and entered as the predictor in logistic regression models for MetS. We compared model fit by R^2^ and selected the group with the largest R^2^ as the primary cluster for subsequent analyses.

***Calculation of cluster:***

Given a pathway matrix $P$ with dimensions *m×n*, a weighted transformation is applied, where each element $P_{ij}$(excluding the first element of each row) is multiplied by the first element of its corresponding row $P_{i1}$. This transformation can be expressed mathematically as:

$\mathbf{W}_{\mathbf{ij}}\mathbf{=}\mathbf{P}_{\mathbf{ij}}\boldsymbol{\times}\mathbf{P}_{\mathbf{i1}}$**.**

The first element of each row $P_{i1}$​ represents the beta value returned from the linear regression of the bacteria in that row with the X variable.

After the transformation, the sum of the weighted elements for each row is calculated, excluding the first element, resulting in a vector $b$. Each component $b_{i}$ of this vector represents the sum of the weighted elements for row *i*, calculated as:

$\mathbf{b}_{\mathbf{i}}\mathbf{=}\sum_{\mathbf{j}\mathbf{=}\mathbf{2}}^{\mathbf{n}} \mathbf{W}_{\mathbf{ij}}$**.**

This process can be summarized by the following formula:

$$\mathbf{b}_{\boldsymbol{i}}\mathbf{=}\sum_{\boldsymbol{j}\mathbf{=}\boldsymbol{2}}^{\boldsymbol{n}} \left( \boldsymbol{P}_{\boldsymbol{ij}}\boldsymbol{\times}\boldsymbol{P}_{\boldsymbol{i}\boldsymbol{1}} \right) \boldsymbol{\forall}\boldsymbol{i}\boldsymbol{\in\{}\boldsymbol{1}\boldsymbol{,\ldots,}\boldsymbol{m}\boldsymbol{\}}$$

The resulting vector $b$ captures the total weighted values for each row, providing a summarized representation of the pathway matrix's weighted transformation[15].

After calculate the cluster value of bacteria related to HLS and bring it into mediation analysis to MetS. Mediation analysis using the mediate function from the R package mediation V4.5.0. We estimated the total effect, direct effect and indirect effect of cluster from mediation models. To employ a more objective serum biomarker that provides a clearer representation of participants' metabolic dysregulation and the low-grade inflammation associated with metabolic syndrome, we utilized serum creatinine levels, which exhibit a strong correlation with metabolic syndrome and other metabolic disorders[16], as well as inflammatory markers, to further identify key microbial species. Both significantly associated with creatinine and MetS were considered as selected species, after adjusting for age, sex, education level, medication use, and family medical history.

***SNPs selection and bi-directional Mendelian randomization analysis***

A bi-directional one-sample Mendelian randomization (MR） analysis was employed to assess the causal associations between key species and MetS. Quality control was performed with PLINK (v.1.9), and we excluded single-nucleotide polymorphisms (SNPs) with (1) Minor Allele Frequency <5%; (2) Hardy-Weinberg equilibrium violation *P* <0.00001, and (3) genotype calling rate <5%.^13^ Furthermore, we calculated linkage disequilibrium (LD) between each pair of SNPs at a window of 50 SNPs, and removed one of a pair of SNPs if LDs was higher than 0.5. Finally, a total of 1543256 SNPs were retained in the current genome-wide association study (GWAS) with bacterial species among 1,342 participants. The threshold of *P*<1×10^-5^ were set for identifying SNPs associated with microbiota or metabolites to maximize the amount of genetic variance explained by selected SNPs, as previously described[17].

Bidirectional MR analysis was performed by MendelianRandomization[18] (version 0.9.0). SNPs with F statistic (beta^2^/SE^2^) > 10 were considered as strong genetic instrumental variables (IVs) and used for subsequent MR analysis. MR estimates were calculated using inverse-variance-weighted (IVW) methods. We used IVW as the primary estimator. For each SNP, we computed a wald ratio and combined ratios across instruments using a weighted regression through the origin, with weights. Under the standard MR assumptions and in the absence of directional horizontal pleiotropy, IVW yields an unbiased and most precise causal estimate. In addition, we report MR estimates using simple median, and the MR-Egger regression methods. To ensure the validity of the results, several sensitivity analyses were performed: 1) check out the horizontal pleiotropy (MR-Egger intercept *P* > 0.05); 2) check out the heterogeneity (Cochran’s Q-test *P* > 0.05). In addition, to identify potential heterogeneous SNPs, the “leave-one-out” analysis was performed by omitting each instrumental SNP in turn. Furthermore, we performed reverse MR analysis on selected species by the same analysis procedure to ensure its causality.

***Identification of microbial metabolites***

As the metabolomics data were extremely skewed, metabolite LC-MS peak areas were log transformed and scaled to a mean of zero and SD of one before analysis. First, general linear regression models were used to evaluate the independent associations between metabolites and the HLS. Metabolites significantly associated with HLS were then selected for further analyses. Second, logistic regression analyses, adjusted for relevant covariates including age, sex, education level, medication use, and family medical history, were performed to identify metabolites significantly associated with MetS. Metabolites that were positively associated with HLS and negatively associated with MetS were considered potentially protective and chosen for the next stage of analysis. Next, the correlations between these selected metabolites and CREA were examined, and metabolites showing significant negative correlations with CREA were retained. The selected metabolites were further analyzed in relation to the selected species and metabolic functions, and these findings were integrated into a biological interpretation framework.

Finally, the selected metabolites identified from the previous analyses were subjected to targeted quantitative analysis using ultra-performance liquid chromatography–tandem mass spectrometry (UPLC–MS/MS) with isotope-labeled internal standards, enabling absolute quantification of their concentrations in plasma. Subsequently, the Wilcoxon rank-sum test was applied to compare the distributions of these metabolites between the MetS and non-MetS groups. Mediation analysis was conducted to determine whether, and to what extent, the identified metabolites mediated the effect of the HLS on MetS.

***Identification of microbial enzymes involved in the production of selected metabolites***

To delineate how *Roseburia hominis*，*Odoribacter splanchnicus* and *Alistipes putredinis* influence the level of betaine and cinnamoylglycine, a BLASTP search was performed with the protein sequence of enzymes known to be involved in homolactic fermentation, and its closely related pathways, as the query against all open reading frames of *Roseburia hominis*, *Odoribacter splanchnicus* and *Alistipes putredinis*. Bacterial protein sequences were annotated against the eggNOG v5.0 database using eggNOG-mapper with DIAMOND (BLASTP), all other parameters were left at their defaults[19].

***In silico analysis for downstream targets of selected metabolites***

The potential downstream target of selected molecules which could mediate the effect of *Roseburia hominis*，*Odoribacter splanchnicus* and *Alistipes putredinis* on MetS, including betaine and cinnamoylglycine, were predicted by Similarity ensemble approach (SEA, https://sea.bkslab.org) and SwissTargetPrediction (http://www.swisstargetprediction.ch). Furthermore, Gene Ontology(GO) enrichment analysis was performed with all those predicted downstream targets as input gene sets using clusterProfiler with org.Hs.eg.db. To ensure the reliability and statistical significance of the results, we implemented stringent filtering criteria. Firstly, we set a *P*-value threshold, considering only enrichment results with *P*-value < 0.05. This filtering condition helped identify statistically significant enrichments of GO terms. Additionally, we applied the Benjamini–Hochberg FDR criterion (q < 0.05), and the top 5 Cellular Component (CC) and Molecular Function (MF) terms were shown. The simplifyEnrichment package was then employed to cluster the similarity matrices of the enriched terms into groups using "binary cuts" to explore the main biological information contained in the results of the enrichment analysis

***Targeted quantification of betaine and cinnamoylglycine by UPLC–MS/MS***

A targeted analysis was carried out by using UPLC-MS/MS method to quantify betaine and cinnamoylglycine in plasma samples. Calibration standard of betaine, cinnamoylglycine were obtained from Sigma-Aldrich (St. Louis, MO, USA). Briefly, 100 µL plasma samples were added to a mixture of 600 µL of acetonitrile: methanol (75:25, v/v) including 2 internal standards, Betaine-d9 at 10umol/L (Alta Scientific Co., Ltd, Tianjin, China), [2,2-2H2]-N-trans-Cinnamoylglycine (CMG-d2) at 50 ng/mL (Alta Scientific Co., Ltd, Tianjin, China). The mixture was then vortexed on maximum speed for 5 min at room temperature, sonicated for 5 min with ice for complete protein precipitation and then centrifuged at 15000 ×g for 10 min at 4 °C. The resulting supernatants (600 µL) were transferred to new micro tubes and evaporated to dryness in a water bath (35°C) under nitrogen gas for 2 hours. The dried residues were reconstituted in 100 µL 50% acetonitrile in water and agitated for 5 min, followed by centrifugation at 15,000×g for 10 min at 4 °C. Finally, 90 μL of each sample was recovered and transferred into the autosampler vial, and 1 μL of aliquots was injected into the UPLC–MS/MS system. Chromatographic separation was performed by gradient elution on a Poroshell 120, EC-C18, 2.1 × 100 mm, 2.7 µ column (Agilent) with a UHPLC EC-C18 2.1 × 5 mm ID, 2.7 µ guard cartridge (Agilent). Mobile phase A was 0.1% formic acid and 5 mmol/L ammonium acetate in water, and mobile phase B was 0.1% formic acid in acetonitrile. The metabolites were detected using a Shimadzu Nexera Series system (Shimadzu Corporation, Kyoto, Japan) coupled with an AB Sciex Triple Quad™ 7500 system (Applied Biosystems Inc., Foster City, CA). The temperatures of the column and autosampler were maintained at 35°C and 15°C, respectively. Quantified detection was performed in a multiple-reaction monitoring mode. Gradient elution was programmed as follows: 0-2 min maintained at 10% B, 2.01-4 min linearly increase from 10% to 20% B, 4.01-7 min linearly increase from 20% to 35% B, 7.01-8 min linearly increase from 35% to 90% B, and maintained at 90% B for 2.5 min. The column was equilibrated for 2.49 min with 10% B before the next run. In chromatographic separation 1 µL of sample/standard was used and the flow rate was set at 0.4 mL/min. Electrospray ionization of cinnamoylglycine and betaine were performed in positive mode. The mass-to-charge ratios of the precursor-to-product ion reactions monitored were 206.1→131.0 for cinnamoylglycine, 118.1→59.3 for betaine, 208.1→131 for CMG-d2, 127.3→68.3 for Betaine-d9. The retention times of betaine, betaine-d9, cinnamoylglycine, CMG-d2 were approximately 8.9, 8.9, 4.8 and 4.8 min. The linear range for plasma cinnamoylglycine and betaine standard curves were 0 to 100 ng/mL, and 1.95 to 1000 ng/mL, respectively. A calibration curve was constructed by plotting the peak area ratios of each analyte to the internal standard versus the spiked concentrations. The calibration equations were assessed using the weighted linear regression analysis with a weighing factor of 1/x. The correlation coefficient (r2) should be at least 0.99, and criteria for the calibration standards used in calibration curve was 100 ± 15 % accuracy (relative error) of the nominal concentration.

Altogether, cinnamoylglycine and betaine were measured by a targeted method in 1,342 plasma samples. Due to the large sample size of 1,342, the quantification of cinnamoylglycine and betaine were completed in 7 batches, following the same testing and sample preprocessing methods. To verify data reliability, the combat function of the "sva" embedded in R package was applied to remove the batch effect and merged them into a complete dataset[20]. The distribution of 7 batches before and after calibration for batch effects was then evaluated with principal component analysis based on R package “FactoMineR”

# Supplementary Results

**Supplemental Figure 1**

1423 participants aged 31-78 with fecal collection

**1342 in final analysis**

Participants with complete fecal and blood samples, phenotype data.

**Exclusion**:

1. Diagnosis with pulmonary tuberculosis, chronic hepatitis or severe renal insufficiency (n=37)
2. Application of antibiotic (n=7)
3. Lack of information on diagnosis of metabolic syndrome (n=37)

**Microbiome Features**

Mediating effect of core species.

Functional pathways selected.

**Plasma metabolites**

Key plasma metabolites identified.

**Integrative Analysis**

1. How the gut microbiome changes with lifestyle and MetS.
2. Key effectors of gut microbiota.

# Supplemental Figure 1. Flow chart for study participant recruitment and data analysis.

**
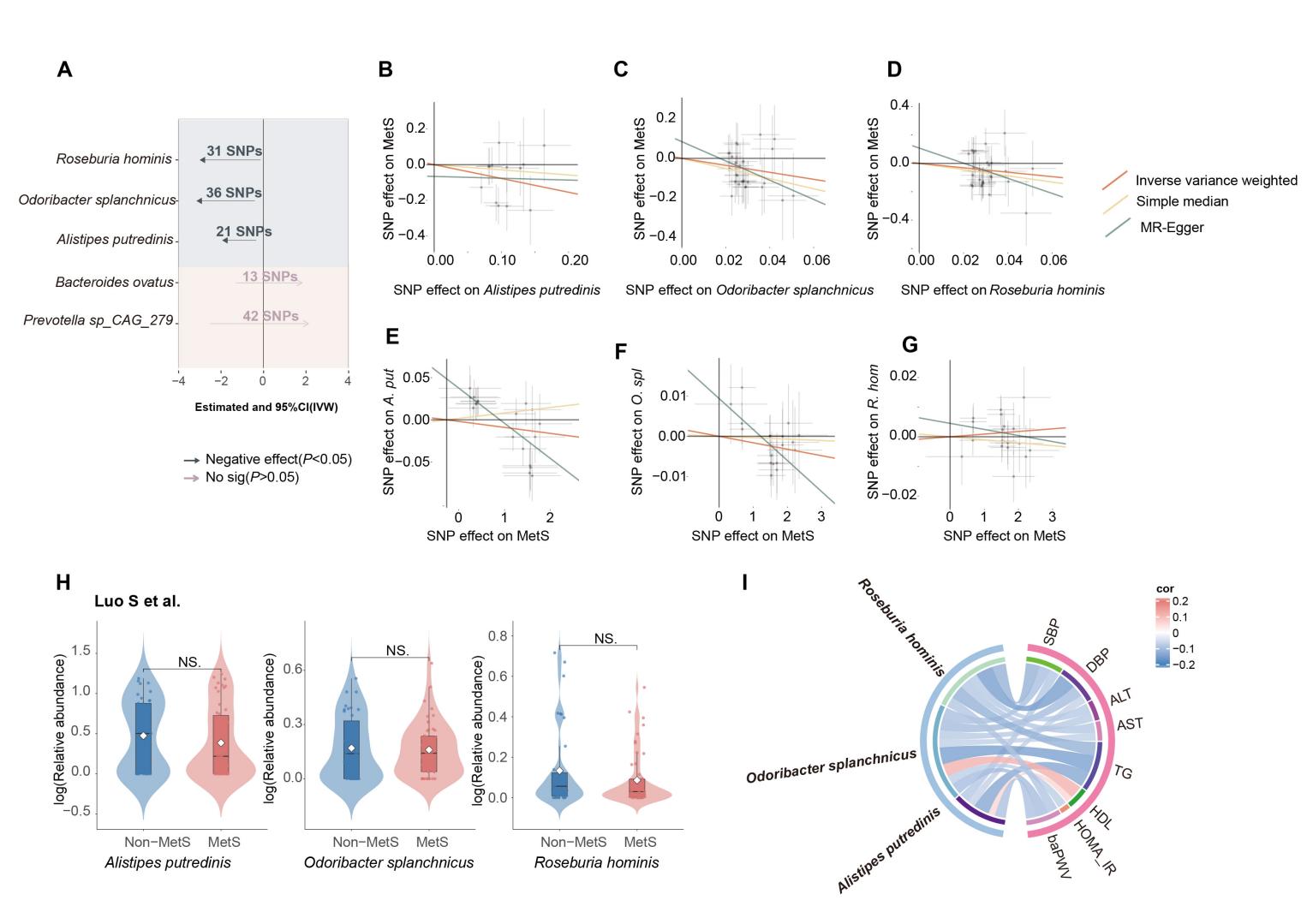
Supplemental Figure 2**

# Supplemental Figure 2 HLS-related species are linked to MetS.

(A) The forest plot showing the MR associations observed between driver species and MetS. Data were presented as the Beta value with corresponding 95% confidence interval. (B-D) Scatterplot of associations between genetic variants and *Alistipes putredinis*, *Odoribacter splanchnicus* and *Roseburia hominis* versus between genetic variants and MetS. (E-G) Scatterplot of associations between genetic variants and MetS versus between genetic variants and *Alistipes putredinis*, *Odoribacter splanchnicus* and *Roseburia hominis.* For graphical representation, *Alistipes putredinis,* *Odoribacter splanchnicus* and *Roseburia hominis*, were abbreviated as *A.put*, *O.spl*, and *R.hom*, respectively. (H) The relative abundance of *Alistipes putredinis*, *Odoribacter splanchnicus* and *Roseburia hominis* in subjects with or without MetS in validation cohorts from China. (I) Chord diagram visualizing the spearman’s correlation coefficients between negatively MetS-related bacterial species and clinical parameters. Positive correlations are shown in red, while negative correlations are shown in blue(*P*_adj_ <0.05).

SBP: systolic blood pressure; DBP: diastolic blood pressure; TG: total cholesterol; HDL: high-density lipoprotein cholesterol; baPWV: brachial-ankle pulse wave velocity; HOMA-IR: Homeostatic model assessment of insulin resistance; IVW: inverse-variance weighted.

**Supplemental Figure 3**

**
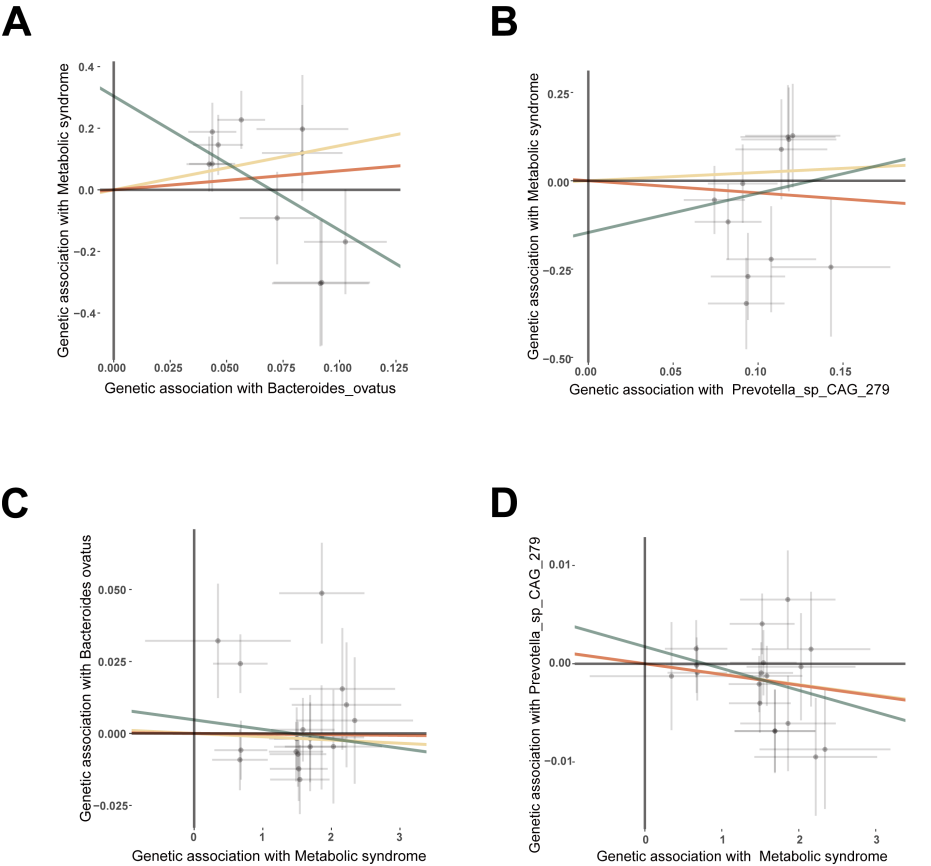
**

Supplemental Figure 3. The relative abundance of species. (A-B) Scatterplot of associations between genetic variants and Bacteroides ovatus, Prevotella_sp_CAG_279 versus between genetic variants and MetS. (C-D) Scatterplot of associations between genetic variants and MetS versus between genetic variants and Bacteroides ovatus and Prevotella_sp_CAG_279.

**
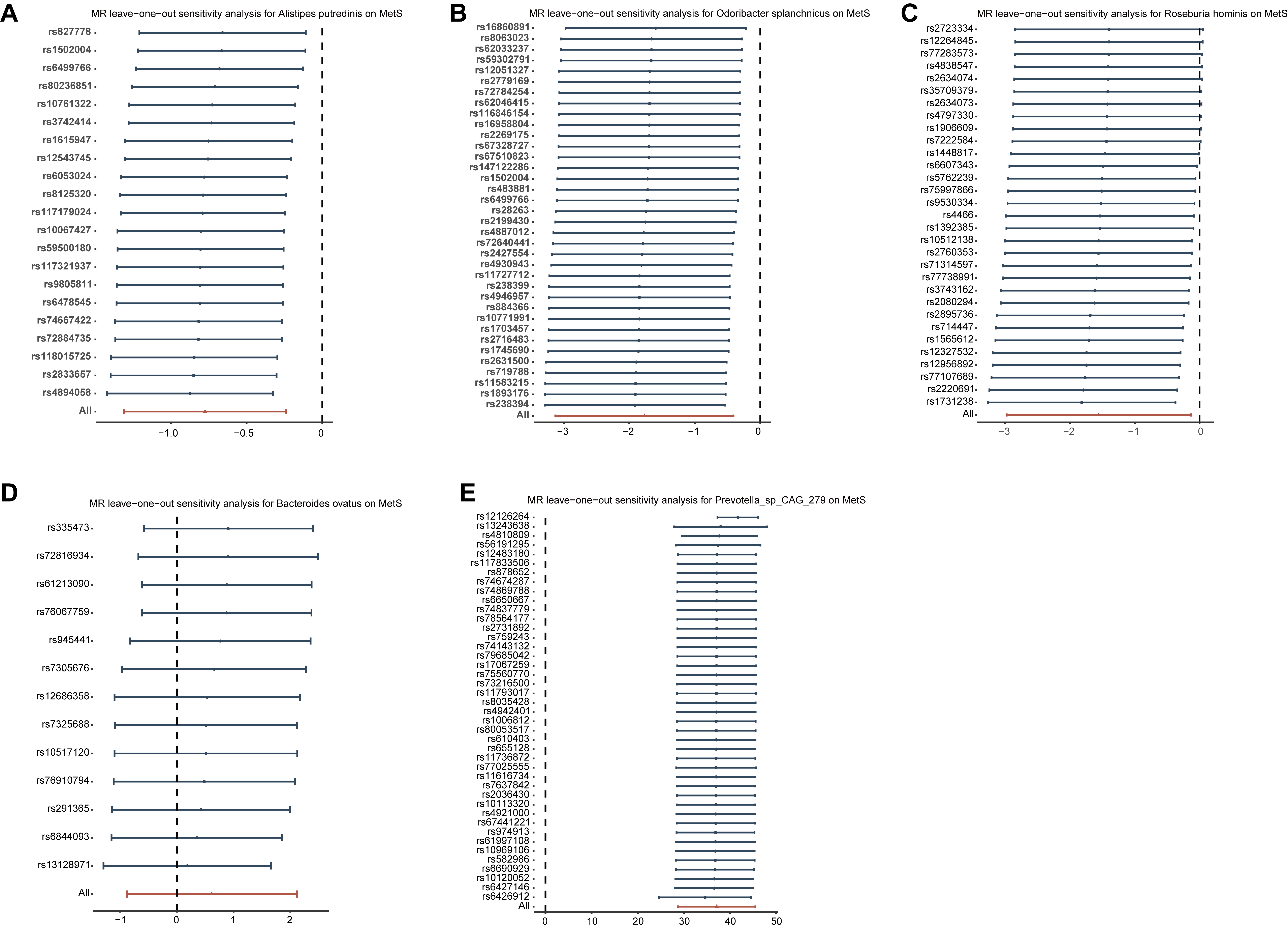
Supplemental Figure 4**

Supplemental Figure 4. Leave-one-out plots for the causal association between Roseburia hominis, Odoribacter splanchnicus, Alistipes putredinis, Prevotella sp_CAG_279, Bacteroides ovatus and MetS. Leave-one-out analysis for (A) *Alistipes putredinis* (B) *Odoribacter splanchnicus* (C) *Roseburia hominis*(D) *Bacteroides ovatus(*E*) Prevotella_sp_CAG_279*. Each row represented the SNP-exposure effect size with corresponding SE. The red lines represented the average effect of all.

**Supplemental Figure 5**

#
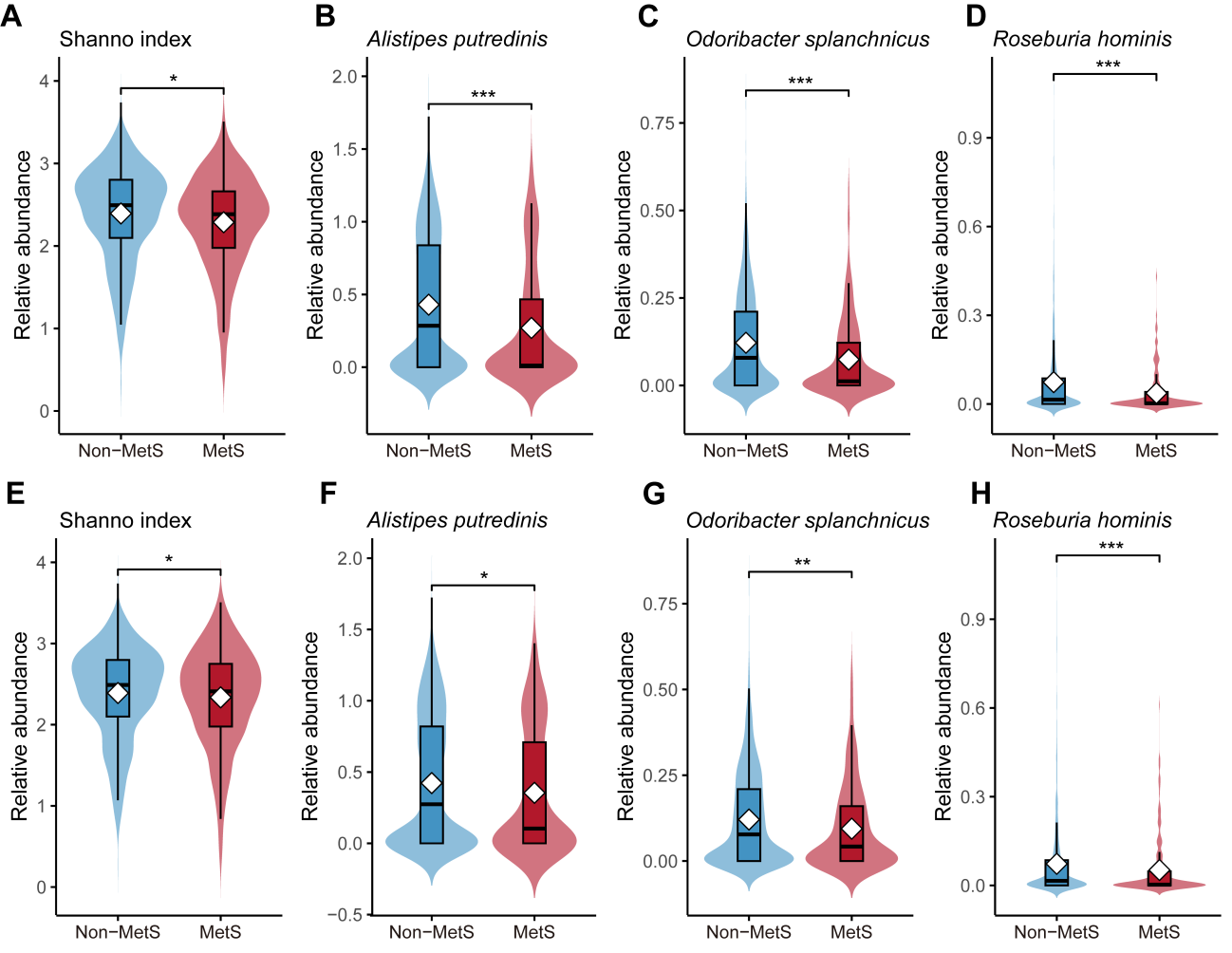
Supplemental Figure 5. Differences in gut microbial diversity and species between MetS and Non‑MetS groups classified by CDS and IDF criteria.

Figure (A–D) are based on the CDS criteria, and panels (E–H) are based on the IDF criteria. Violin plots display the distribution of values with overlaid box plots (median and interquartile range) and a white diamond indicating the mean. Specifically, A and E show the Shannon index of alpha diversity; B and F show the relative abundance of *Alistipes putredinis*; C and G show the relative abundance of *Odoribacter splanchnicus*; and D and H show the relative abundance of *Roseburia hominis*. Statistical significance is indicated above each comparison, with asterisks denoting levels: *P* values were determined by Wilcoxon rank-sum test, with ^*^*P*<0.05, ^**^*P*<0.01, ^***^*P*<0.001.

**Supplemental Figure 6**


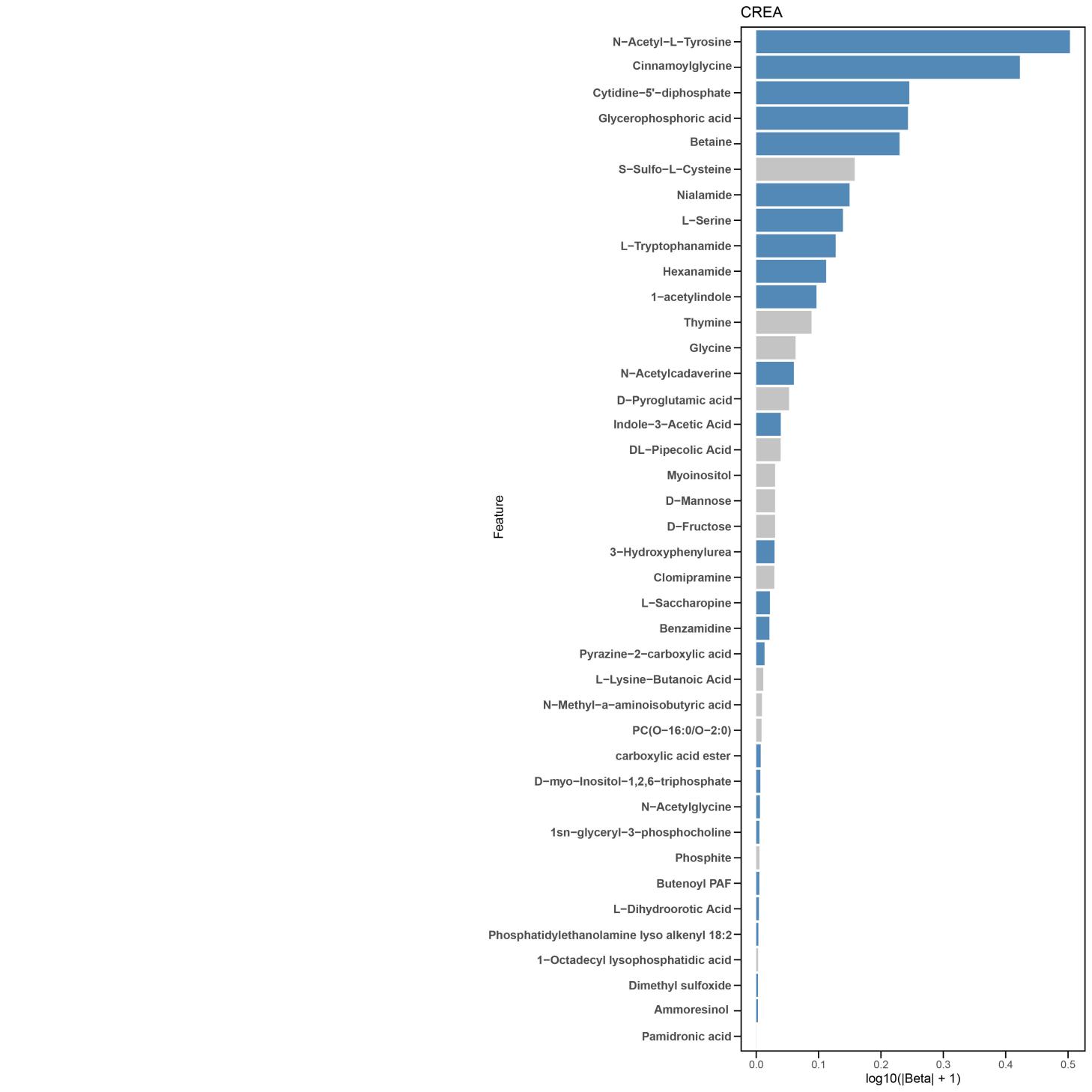
 Supplemental Figure 6. The independent association of metabolites with creatinine(CREA).The model adjusted for age, sex, education level, medication use, and family medical history. Red, blue and gray indicate positive, negative and insignificant correlations with MetS. *P* values were determined by linear regression (*P*_adj_ < 0.05).

**Supplemental Figure 7**

# Supplemental Figure
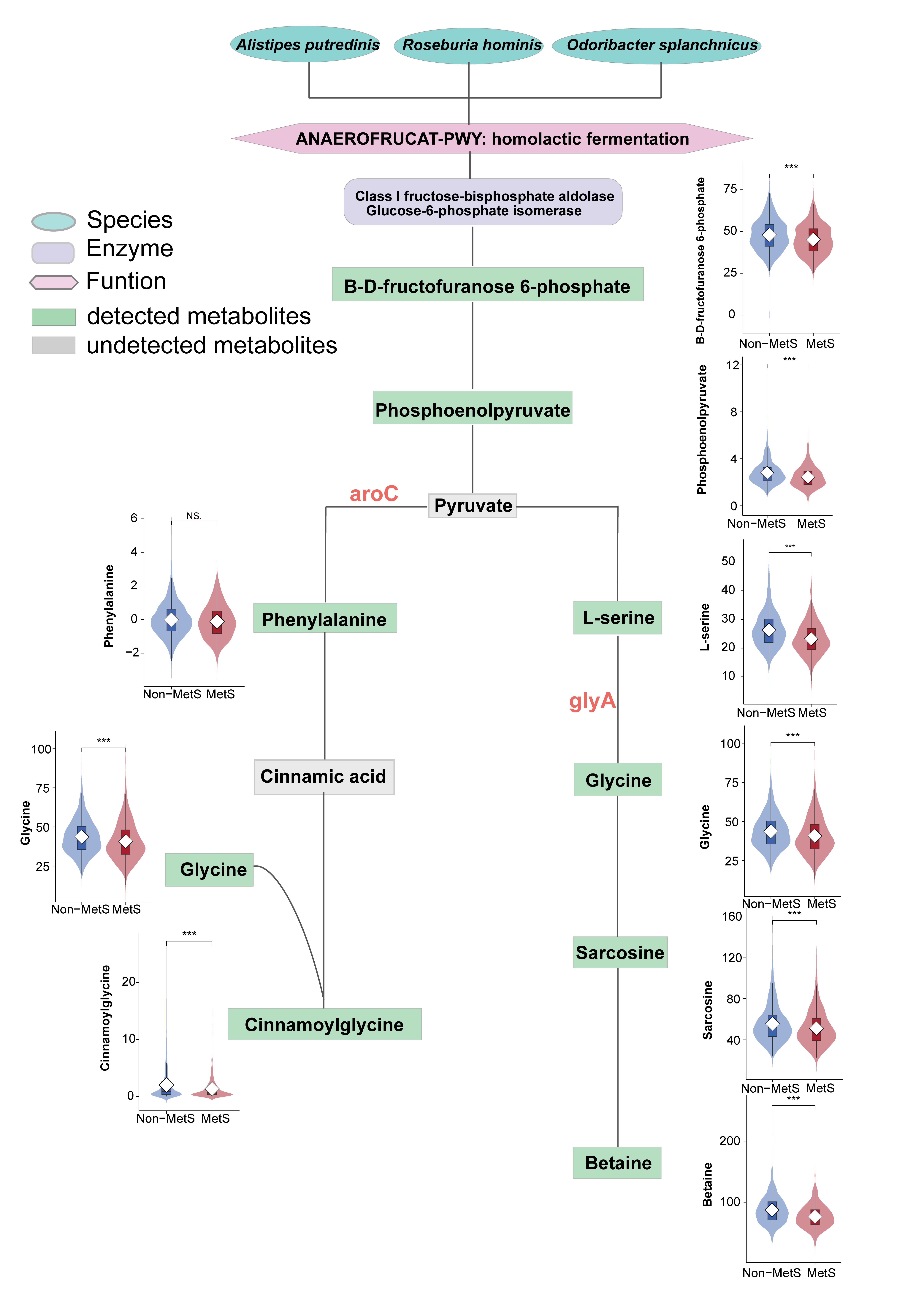
7. Microbial metabolites serve as effectors to MetS.

Illustration of how *Alistipes putredinis*, *Odoribacter splanchnicus*, and *Roseburia hominis*, as well as enhanced hormone fermentation, and subsequent increased production of betaine and cinnamoylglycine, contribute to the protection against MetS. Grey, red, and blue indicate undetected, MetS-related, and non-MetS-related species/functions/metabolites in individuals. *P* values were determined by Wilcoxon rank-sum test, with ^*^*P*< 0.05, ^**^*P*<0.01, ^***^*P*<0.001.

**
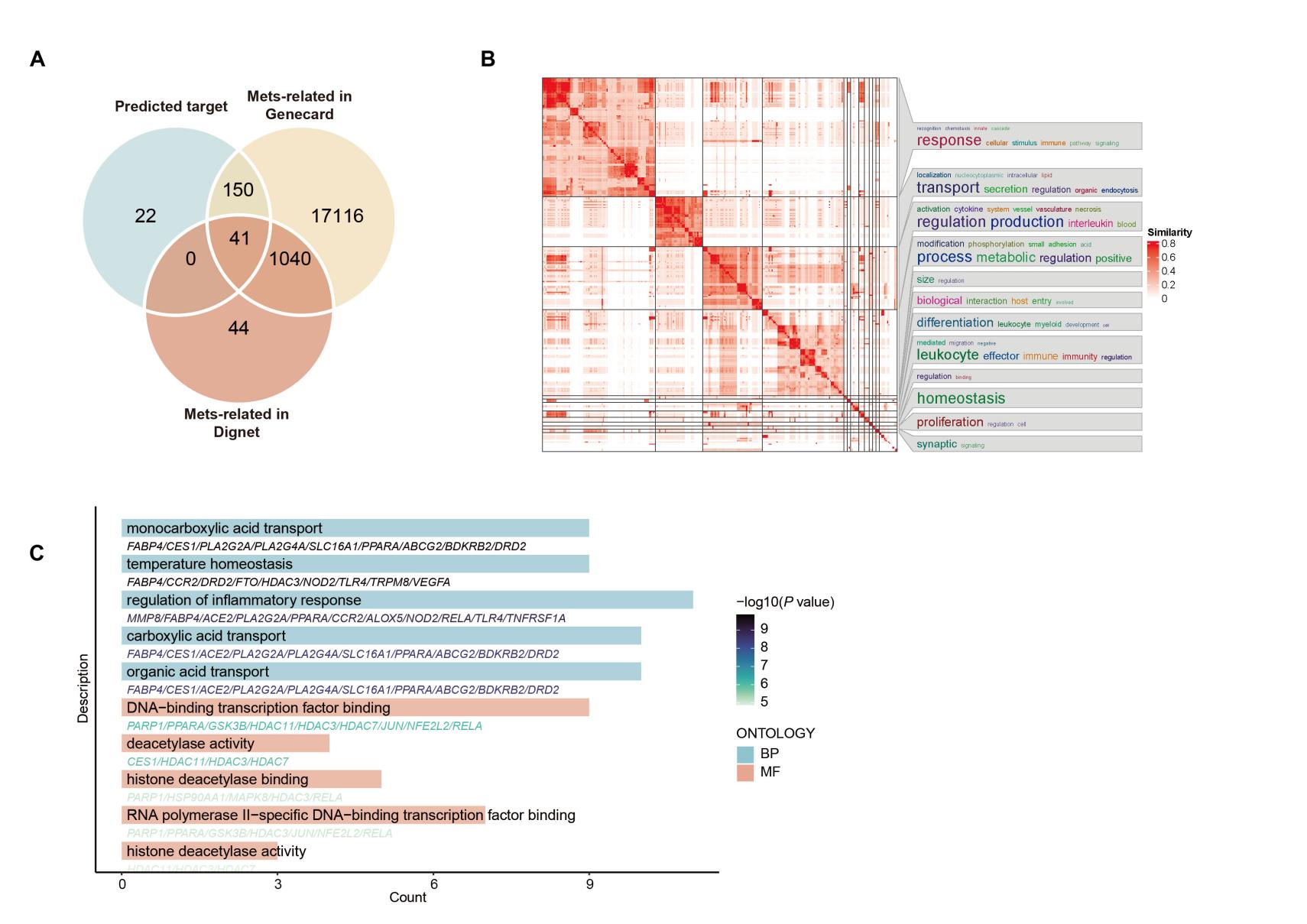
Supplemental Figure 8**

# Supplemental Figure 8. Predicted targets and enriched pathways of key metabolites and MetS.

(A) Overlap of potential downstream targets of the betaine and cinnamoylglycine and genes related to MetS in Dignet and GeneCards repositories, respectively.

(B) Similarity clustering heatmap for enriched pathways with term frequencies exhibited by font size.

(C) Top 5 enriched biological process **(**BP**)** and molecular function **(**MF**)** related to metabolic syndrome pathway.

# Supplemental Table 1. MetaCyc ID and the corresponding names of each pathway

| **MetaCyc ID** | **F**unction name |
| --- | --- |
| ALLANTOINDEG-PWY | superpathway of allantoin degradation in yeast |
| ANAEROFRUCAT-PWY | homolactic fermentation |
| ARGDEG-PWY | superpathway of L-arginine, putrescine, and 4-aminobutanoate degradation |
| ARGORNPROST-PWY | arginine, ornithine and proline interconversion |
| AST-PWY | L-arginine degradation II (AST pathway) |
| CENTFERM-PWY | pyruvate fermentation to butanoate |
| ECASYN-PWY | enterobacterial common antigen biosynthesis |
| HCAMHPDEG-PWY | 3-phenylpropanoate and 3-(3-hydroxyphenyl)propanoate degradation to 2-oxopent-4-enoate |
| ORNARGDEG-PWY | superpathway of L-arginine and L-ornithine degradation |
| ORNDEG-PWY | superpathway of ornithine degradation |
| P108-PWY | pyruvate fermentation to propanoate I |
| P163-PWY | L-lysine fermentation to acetate and butanoate |
| P461-PWY | hexitol fermentation to lactate, formate, ethanol and acetate |
| PWY-241 | C4 photosynthetic carbon assimilation cycle, NADP-ME type |
| PWY-4321 | L-glutamate degradation IV |
| PWY-5030 | L-histidine degradation III |
| PWY-5509 | adenosylcobalamin biosynthesis from cobyrinate a,c-diamide I |
| PWY-5675 | nitrate reduction V (assimilatory) |
| PWY-5677 | succinate fermentation to butanoate |
| PWY-5723 | Rubisco shunt |
| PWY-5840 | superpathway of menaquinol-7 biosynthesis |
| PWY-5855 | ubiquinol-7 biosynthesis (prokaryotic) |
| PWY-5856 | ubiquinol-9 biosynthesis (prokaryotic) |
| PWY-5857 | ubiquinol-10 biosynthesis (prokaryotic) |
| PWY-5941 | glycogen degradation II (eukaryotic) |
| PWY-6071 | superpathway of phenylethylamine degradation |
| PWY-6143 | CMP-pseudaminate biosynthesis |
| PWY-6590 | superpathway of Clostridium acetobutylicum acidogenic fermentation |
| PWY-6595 | superpathway of guanosine nucleotides degradation (plants) |
| PWY-6690 | cinnamate and 3-hydroxycinnamate degradation to 2-oxopent-4-enoate |
| PWY-6708 | ubiquinol-8 biosynthesis (prokaryotic) |
| PWY-6803 | phosphatidylcholine acyl editing |
| PWY-6837 | fatty acid beta-oxidation V (unsaturated, odd number, di-isomerase-dependent) |
| PWY-7094 | fatty acid salvage |
| PWY-7254 | TCA cycle VII (acetate-producers) |
| PWY-7269 | NAD/NADP-NADH/NADPH mitochondrial interconversion (yeast) |
| PWY-7282 | 4-amino-2-methyl-5-phosphomethylpyrimidine biosynthesis (yeast) |
| PWY-7409 | phospholipid remodeling (phosphatidylethanolamine, yeast) |
| PWY-7456 | mannan degradation |
| PWY0-1277 | 3-phenylpropanoate and 3-(3-hydroxyphenyl)propanoate degradation |
| PWY0-1338 | polymyxin resistance |
| PWY0-41 | allantoin degradation IV (anaerobic) |
| PWY0-845 | superpathway of pyridoxal 5'-phosphate biosynthesis and salvage |
| PWY66-367 | ketogenesis |
| PYRIDOXSYN-PWY | pyridoxal 5'-phosphate biosynthesis I |
| UBISYN-PWY | superpathway of ubiquinol-8 biosynthesis (prokaryotic) |

# Supplemental Table 2. Baseline characteristics of the study participants in discovery and validation cohort

| **Characteristics** | **Validation cohort** | **Discovery cohort** | **Total** | ***P* value** |
| --- | --- | --- | --- | --- |
|  | (N=89) | (N=1342) | (N=1431) |  |
| Male, n (%) | 39 (43.82) | 607 (45.23) | 646 (45.14) | 0.796 |
| Age, year | 52.00 (47.00-58.00) | 50.00 (45.00-56.00) | 50.00 (45.00;57.00) | 0.094 |
| SBP, mm Hg | 124.00 (114.50-135.50) | 124.00 (114.00-136.00) | 124.00 (114.00;135.75) | 0.967 |
| DBP, mm Hg | 80.50 (72.50-88.00) | 80.50 (74.00-88.00) | 80.50 (74.00;88.00) | 0.591 |
| BMI | 23.80 (21.00-26.10) | 23.91 (21.71-26.14) | 23.89 (21.70;26.14) | 0.252 |
| Waist circumference, cm | 82.20 (74.10-88.60) | 84.00 (76.80-91.00) | 84.00 (76.50;90.95) | 0.040 |
| MetS (%) | 12 (13.48) | 334 (24.89) | 346 (24.18) | 0.015 |
| Fasting glucose, mmol/L | 4.32 (4.04-4.70) | 4.70 (4.28-5.20) | 4.68 (4.25;5.18) | < 0.001 |
| TG, mmol/L | 0.98 (0.75-1.30) | 1.18 (0.84-1.70) | 1.16 (0.83;1.68) | 0.001 |
| TC, mmol/L | 4.96 (4.53-5.39) | 5.27 (4.67-5.94) | 5.23 (4.67;5.91) | 0.001 |
| HDL-c, mmol/L | 2.94 (2.58-3.42) | 3.16 (2.67-3.68) | 3.14 (2.66;3.65) | 0.022 |
| LDL-c, mmol/L | 1.36 (1.19-1.56) | 1.37 (1.16-1.62) | 1.37 (1.16;1.62) | 0.815 |

Data were expressed as median (interquartile range) or n (%), and *P* Value were determined by Wilcoxon rank-sum test, χ^2^ test or Fisher exact test, as appropriate. SBP： systolic blood pressure; DBP： diastolic blood pressure; TC： total cholesterol; HDL-c： high-density lipoprotein cholesterol; LDL-c： low-density lipoprotein cholesterol.

# Supplemental Table 3. Baseline characteristics according to the CDS

|  | **Non-MetS** | **MetS** | **Total** | ***P* value** |
| --- | --- | --- | --- | --- |
|  | (N=1103) | (N=239) | (N=1342) |  |
| Female, n(%) | 586 (53.13) | 149 (62.34) | 735 (54.77) | 0.009 |
| Age, year | 50.00 (44.00-56.00) | 52.00 (47.00-60.00) | 50.00 (45.00-56.00) | < 0.001 |
| Education |  |  |  |  |
| Below high school, n (%) | 751 (68.09) | 194 (81.17) | 945 (70.42) | < 0.001 |
| High school and above, n (%) | 352 (31.91) | 45 (18.83) | 397 (29.58) | < 0.001 |
| BMI, kg/m2 | 23.39 (21.31-25.40) | 26.82 (25.25-29.02) | 23.91 (21.71-26.14) | < 0.001 |
| Waist circumference, cm | 81.70 (75.65-89.00) | 94.00 (86.00-97.00) | 84.00 (76.80-91.00) | < 0.001 |
| Hip circumference, cm | 93.60 (90.00-97.60) | 99.30 (95.30-103.85) | 94.50 (90.60-99.00) | < 0.001 |
| WHR | 0.88 (0.83-0.92) | 0.93 (0.89-0.97) | 0.89 (0.84-0.93) | < 0.001 |
| **Comorbidities** |  |  |  |  |
| Diabetes, n (%) | 16 (1.59) | 48 (14.37) | 64 (4.77) | < 0.001 |
| Hypertension, n (%) | 144 (13.06) | 83 (34.73) | 227 (16.92) | < 0.001 |
| **Medication** |  |  |  |  |
| Antidiabetic, n (%) | 22 (1.99) | 32 (13.39) | 54 (4.02) | < 0.001 |
| Lipid lowering, n (%) | 28 (2.54) | 14 (5.86) | 42 (3.13) | 0.008 |
| Antihypertensive, n (%) | 125 (11.33) | 76 (31.80) | 201 (14.98) | < 0.001 |
| **Healthy Lifestyle factors** |  |  |  |  |
| Smoking status |  |  |  |  |
| Never, n (%) | 850 (77.06) | 189 (79.08) | 1039 (77.42) | 0.008 |
| Daily, n (%) | 183 (16.59) | 25 (10.46) | 208 (15.50) | 0.008 |
| former, n (%) | 70 (6.35) | 25 (10.46) | 95 (7.08) | 0.008 |
| Good diet quality, n (%) | 431 (39.08) | 87 (36.40) | 518 (38.60) | 0.441 |
| High physical activity, n (%) | 361 (32.73) | 74 (30.96) | 435 (32.41) | 0.597 |
| No heavy drinking, n (%) | 1010 (91.57) | 225 (94.14) | 1235 (92.03) | 0.183 |
| **Laboratory measures** |  |  |  |  |
| SBP, mm Hg | 121.50 (112.50-132.50) | 135.00 (127.00-146.50) | 124.00 (114.00-136.00) | < 0.001 |
| DBP, mm Hg | 79.50 (73.50-86.50) | 86.00 (78.75-94.50) | 80.50 (74.00-88.00) | < 0.001 |
| TG, mmol/L | 1.09 (0.80-1.50) | 1.96 (1.35-2.61) | 1.18 (0.84-1.70) | < 0.001 |
| TC, mmol/L | 5.24 (4.65-5.92) | 5.36 (4.94-6.06) | 5.27 (4.67-5.94) | 0.026 |
| HDL-c, mmol/L | 1.42 (1.21-1.68) | 1.17 (1.02-1.32) | 1.37 (1.16-1.62) | < 0.001 |
| LDL-c, mmol/L | 3.12 (2.63-3.64) | 3.33 (2.83-3.83) | 3.16 (2.67-3.68) | 0.001 |
| FPG, mmol/L | 4.65 (4.23-5.07) | 5.17 (4.53-5.93) | 4.70 (4.28-5.20) | < 0.001 |
| Creatinine, mmol/L | 74.70 (64.15-88.40) | 73.60 (63.30-87.95) | 74.50 (63.90-88.30) | 0.455 |

Values are n (%), or median (interquartile range).

BMI：Body mass index; HOMA-IR：Homeostatic model assessment of insulin resistance; TG: Triglyceride; TC:Total cholesterol- HDL-c: high-density lipoprotein cholesterol; LDL-c: low-density lipoprotein cholesterol; TyG: triglyceride-glucose index; FPG: Fasting plasma glucose; WHR: Waist-to-Hip Ratio.

**Supplemental Table 4. Baseline characteristics according to the IDF**

|  | **Non-MetS** | **MetS** | **Total** | ***P* value** |
| --- | --- | --- | --- | --- |
|  | (N=1179) | (N=163) | (N=1342) |  |
| Female, n(%) | 681 (57.76) | 54 (33.13) | 735 (54.77) | < 0.001 |
| Age, year | 50.00 (44.00-56.00) | 51.00 (46.50-58.50) | 50.00 (45.00-56.00) | 0.011 |
| Education |  |  |  |  |
| Below high school, n (%) | 812 (68.87) | 133 (81.60) | 945 (70.42) | 0.001 |
| High school and above, n (%) | 367 (31.13) | 30 (18.40) | 397 (29.58) | 0.001 |
| BMI, kg/m2 | 23.48 (21.45-25.45) | 27.14 (25.82-29.40) | 23.91 (21.71-26.14) | < 0.001 |
| Waist circumference, cm | 82.20 (76.00-89.15) | 94.00 (89.85-98.00) | 84.00 (76.80-91.00) | < 0.001 |
| Hip circumference, cm | 93.80 (90.00-98.00) | 99.40 (96.60-104.40) | 94.50 (90.60-99.00) | < 0.001 |
| WHR | 0.88 (0.83-0.92) | 0.94 (0.90-0.97) | 0.89 (0.84-0.93) | < 0.001 |
| **Comorbidities** |  |  |  |  |
| Diabetes, n (%) |  |  |  |  |
| Hypertension, n (%) | 145 (12.30) | 82 (50.31) | 227 (16.92) | < 0.001 |
| **Medication** |  |  |  |  |
| Antidiabetic, n (%) | 22 (1.87) | 32 (19.63) | 54 (4.02) | < 0.001 |
| Lipid lowering, n (%) | 30 (2.54) | 12 (7.36) | 42 (3.13) | 0.001 |
| Antihypertensive, n (%) | 124 (10.52) | 77 (47.24) | 201 (14.98) | < 0.001 |
| **Healthy Lifestyle factors** |  |  |  |  |
| Smoking status |  |  |  |  |
| Never, n (%) | 929 (78.80) | 110 (67.48) | 1039 (77.42) | < 0.001 |
| Daily, n (%) | 182 (15.44) | 26 (15.95) | 208 (15.50) | < 0.001 |
| former, n (%) | 68 (5.77) | 27 (16.56) | 95 (7.08) | < 0.001 |
| Good diet quality, n (%) | 459 (38.93) | 59 (36.20) | 518 (38.60) | 0.501 |
| High physical activity, n (%) | 376 (31.89) | 59 (36.20) | 435 (32.41) | 0.271 |
| No heavy drinking, n (%) | 1090 (92.45) | 145 (88.96) | 1235 (92.03) | 0.123 |
| **Laboratory measures** |  |  |  |  |
| SBP, mm Hg | 122.50 (112.50-132.75) | 140.50 (130.25-148.75) | 124.00 (114.00-136.00) | < 0.001 |
| DBP, mm Hg | 79.50 (73.50-86.00) | 91.50 (84.00-96.75) | 80.50 (74.00-88.00) | < 0.001 |
| TG, mmol/L | 1.10 (0.81-1.51) | 2.26 (1.81-3.12) | 1.18 (0.84-1.70) | < 0.001 |
| TC, mmol/L | 5.25 (4.66-5.90) | 5.43 (4.92-6.21) | 5.27 (4.67-5.94) | 0.012 |
| HDL-c, mmol/L | 1.40 (1.20-1.66) | 1.07 (0.94-1.28) | 1.37 (1.16-1.62) | < 0.001 |
| LDL-c, mmol/L | 3.14 (2.65-3.64) | 3.33 (2.84-3.92) | 3.16 (2.67-3.68) | 0.002 |
| FPG, mmol/L | 4.65 (4.24-5.08) | 5.34 (4.65-6.54) | 4.70 (4.28-5.20) | < 0.001 |
| Creatinine, mmol/L | 73.30 (63.10-87.00) | 83.40 (69.85-98.45) | 74.50 (63.90-88.30) | < 0.001 |

Values are n (%), or median (interquartile range).

BMI：Body mass index; HOMA-IR：Homeostatic model assessment of insulin resistance; TG: Triglyceride; TC:Total cholesterol; HDL-c: high-density lipoprotein cholesterol; LDL-c: low-density lipoprotein cholesterol; TyG: triglyceride-glucose index; FPG: Fasting plasma glucose; WHR: Waist-to-Hip Ratio.

**Supplemental Table 5. Alignment results for bacterial key enzymes related to metabolites**

| **Species** | **Assembly accession** | **Gene** | **EC number** | **K number (KEGG)** | **E-value** | **Bit score** | **Protein accession (RefSeq WP_)** |
| --- | --- | --- | --- | --- | --- | --- | --- |
| *Alistipes putredinis* | GCA_000154465.1 | pgi | 5.3.1.9 | K01810 | 3.69E-175 | 442 | WP_013610982.1 |
|  |  | aroC | 4.2.3.5 | K01736 | 4.77E-247 | 677 | WP_004327790.1 |
|  |  | glyA | 2.1.2.1 | K00600 | 2.12E-311 | 848 | WP_004328002.1 |
| *Odoribacter splanchnicus* | GCA_900187175.1 | fba | 4.1.2.13 | K11645 | 1.81E-268 | 715 | WP_013611026.1 |
|  |  | aroC | 4.2.3.5 | K01736 | 4.50E-259 | 710 | WP_013611136.1 |
|  |  | glyA | 2.1.2.1 | K00600 | 0.0 | 863 | WP_013611848.1 |
| *Roseburia hominis* | GCA_000225345.1 | pgi | 5.3.1.9 | K01810 | 0.0 | 880 | WP_013610982.1 |
|  |  | aroC | 4.2.3.5 | K01736 | 2.62E-261 | 716 | WP_014079402.1 |
|  |  | glyA | 2.1.2.1 | K00600 | 6.94E-300 | 817 | WP_044024681.1 |

# Assembly accessions are from NCBI (GCA), and protein accessions are non-redundant RefSeq WP_ identifiers. E-values are reported in scientific notation; 0.0 indicates values below the reporting threshold of DIAMOND.

# References

1. Yang J, Luo S, Liu Y, Hong M, Qiu X, Lin Y, et al. Cohort Profile: South China Cohort. Int J Epidemiol. 2024 Feb 14;53[2]:dyae028.

2. Matthews DR, Hosker JP, Rudenski AS, Naylor BA, Treacher DF, Turner RC. Homeostasis model assessment: insulin resistance and beta-cell function from fasting plasma glucose and insulin concentrations in man. Diabetologia. 1985 Jul;28[7]:412–9.

3. Conventional and genetic evidence on alcohol and vascular disease aetiology: a prospective study of 500 000 men and women in China. The Lancet. 2019 May 4;393[10183]:1831–42.

4. Craig CL, Marshall AL, Sjöström M, Bauman AE, Booth ML, Ainsworth BE, et al. International physical activity questionnaire: 12-country reliability and validity. Med Sci Sports Exerc. 2003 Aug;35[8]:1381–95.

5. Piercy KL, Troiano RP. Physical Activity Guidelines for Americans From the US Department of Health and Human Services. Circ Cardiovasc Qual Outcomes [Internet]. 2018 Nov;

6. Yin Z, Fei Z, Qiu C, Brasher MS, Kraus VB, Zhao W, et al. Dietary Diversity and Cognitive Function among Elderly People: A Population-Based Study. J Nutr Health Aging. 2017;21[10]:1089–94.

7. Zhang YB, Chen C, Pan XF, Guo J, Li Y, Franco OH, et al. Associations of healthy lifestyle and socioeconomic status with mortality and incident cardiovascular disease: two prospective cohort studies. BMJ. 2021 Apr 14;373:n604.

8. S L, S R, Ge L, S R, Bn M. Risk of Sleep Apnea Is Associated with Abdominal Obesity Among Asian Americans: Comparing Waist-to-Hip Ratio and Body Mass Index. J Racial Ethn Health Disparities. 2024 Feb;11[1].

9. Yao F, Bo Y, Zhao L, Li Y, Ju L, Fang H, et al. Prevalence and Influencing Factors of Metabolic Syndrome among Adults in China from 2015 to 2017. Nutrients. 2021 Dec;13[12]:4475.

10. B L, G C, R Z, D H, L T. Temporal trends in the prevalence of metabolic syndrome among middle-aged and elderly adults from 2011 to 2015 in China: the China health and retirement longitudinal study (CHARLS). BMC Public Health [Internet]. 2021 Feb 6;21[1].

11. Chen F, Dai X, Zhou CC, Li KX, Zhang YJ, Lou XY, et al. Integrated analysis of the faecal metagenome and serum metabolome reveals the role of gut microbiome-associated metabolites in the detection of colorectal cancer and adenoma. Gut. 2022 Jul;71[7]:1315–25.

12. Segata N, Waldron L, Ballarini A, Narasimhan V, Jousson O, Huttenhower C. Metagenomic microbial community profiling using unique clade-specific marker genes. Nat Methods. 2012 Jun 10;9[8]:811–4.

13. Caspi R, Billington R, Keseler IM, Kothari A, Krummenacker M, Midford PE, et al. The MetaCyc database of metabolic pathways and enzymes - a 2019 update. Nucleic Acids Res. 2020 Jan 8;48[D1]:D445–53.

14. H T, S H, S T, Y K, N M, T N, et al. Isolation, Identification, and Synthesis of a New Prenylated Cinnamic Acid Derivative from Brazilian Green Propolis and Simultaneous Quantification of Bioactive Components by LC-MS/MS. J Agric Food Chem . 2019 Jun 11;67[44].

15. Li C, Stražar M, Mohamed AMT, Pacheco JA, Walker RL, Lebar T, et al. Gut microbiome and metabolome profiling in Framingham heart study reveals cholesterol-metabolizing bacteria. Cell. 2024 Apr 11;187[8]:1834-1852.e19.

16. D S, W X, J L, Z Z, P F, R L, et al. Serum Uric Acid to Creatinine Ratio and Risk of Metabolic Syndrome in Patients with Overweight/Obesity. Diabetes Metab Syndr Obes Targets Ther. 2023 Sep 28;16.

17. Boulund U, Bastos DM, Ferwerda B, van den Born BJ, Pinto-Sietsma SJ, Galenkamp H, et al. Gut microbiome associations with host genotype vary across ethnicities and potentially influence cardiometabolic traits. Cell Host Microbe. 2022 Oct 12;30[10]:1464-1480.e6.

18. Yavorska OO, Burgess S. MendelianRandomization: an R package for performing Mendelian randomization analyses using summarized data. Int J Epidemiol. 2017 Dec 1;46[6]:1734–9.

19. J HC, D S, D H, A HP, Sk F, H C, et al. eggNOG 5.0: a hierarchical, functionally and phylogenetically annotated orthology resource based on 5090 organisms and 2502 viruses. Nucleic Acids Res [Internet]. 2019 Aug 1;47[D1].

20. Varma S. Blind estimation and correction of microarray batch effect. PloS One. 2020;15[4]:e0231446.
